# Transcriptomic-Based Classification Identifies Prognostic Subtypes and Therapeutic Strategies in Soft Tissue Sarcomas

**DOI:** 10.3390/cancers17172861

**Published:** 2025-08-30

**Authors:** Miguel Esperança-Martins, Hugo Vasques, Manuel Sokolov Ravasqueira, Maria Manuel Lemos, Filipa Fonseca, Diogo Coutinho, Jorge Antonio López, Richard S. P. Huang, Sérgio Dias, Lina Gallego-Paez, Luís Costa, Nuno Abecasis, Emanuel Gonçalves, Isabel Fernandes

**Affiliations:** 1 Medical Oncology Department, Unidade Local de Saúde de Santa Maria, 1649-028 Lisboa, Portugal; sergiodias@medicina.ulisboa.pt (S.D.); luis.costa@ulssm.min-saude.pt (L.C.); 2 Gulbenkian Institute for Molecular Medicine, 1649-035 Lisboa, Portugal; dcoutinho@medicina.ulisboa.pt (D.C.);; 3 Faculdade de Medicina da Universidade de Lisboa, Universidade de Lisboa, 1649-190 Lisboa, Portugal; hugovasques@sapo.pt (H.V.); nunoabecasis@sapo.pt (N.A.); 4 General Surgery Department, Instituto Português de Oncologia de Lisboa Francisco Gentil, 1099-023 Lisboa, Portugal; ffonseca@iposlisboa.min-saude.pt; 5 Instituto de Engenharia de Sistemas e Computadores–Investigação e Desenvolvimento (INESC-ID), 1000-029 Lisboa, Portugal; manuel.sokolov.ravasqueira@tecnico.ulisboa.pt (M.S.R.); emanuel.v.goncalves@tecnico.ulisboa.pt (E.G.); 6 Instituto Superior Técnico, Universidade de Lisboa, 1049-001 Lisboa, Portugal; 7 Pathology Department, Instituto Português de Oncologia de Lisboa Francisco Gentil, 1099-023 Lisboa, Portugal; lemosmaria@hotmail.com; 8 F. Hoffmann-LaRoche AG, 4070 Basel, Switzerland; jorge_antonio.lopez@roche.com; 9 Foundation Medicine Inc., Boston, MA 02141, USA; rhuang@foundationmedicine.com; 10 Medical Oncology Department, Hospital CUF Descobertas, 1998-018 Lisboa, Portugal; fernandescristina@hotmail.com; 11 EpiDoC, CHRC, Nova Medical School, Universidade Nova de Lisboa, 1150-190 Lisboa, Portugal

**Keywords:** soft tissue sarcomas, DNA-seq, RNA-seq, unsupervised machine learning, consensus clustering, prognosis, SARCULATOR, CINSARC, therapeutic targets, precision treatment

## Abstract

**Background: **Soft tissue sarcomas (STSs) histopathological classification system and the clinical and molecular-based tools that are currently employed to estimate its prognosis have several limitations, impacting prognostication and treatment. Clinically driven molecular profiling studies may cover these gaps and offer alternative tools with superior prognostication capability and enhanced precision and personalized treatment approaches identification ability. **Materials and Methods/Results:** We performed DNA sequencing (DNA-seq) and RNA sequencing (RNA-seq) to portray the molecular profile of 102 samples of high-grade STS, comprising the three most common STS histotypes. The analysis of RNA-seq data using unsupervised machine learning models revealed previously unknown molecular patterns, identifying four transcriptomic subtypes/clusters (TCs). This TC-based classification has a clear prognostic value (in terms of overall survival (OS) and disease-free survival (DFS)), a finding that was externally validated using independent patient cohorts. The prognostic value of this TC-based classification outperforms the prognostic accuracy of clinical-based (SARCULATOR nomograms) and molecular-based (CINSARC) prognostication tools, being one of the first molecular-based classifications capable of predicting OS in STS. The analysis of DNA-seq data from the same cohort revealed numerous and, in some cases, never documented molecular targets for precision treatment across different transcriptomic subtypes. The functional and predictive value of each genomic variant was analyzed using the Molecular Tumor Board Portal. **Conclusions:** This newly identified TC-based classification offers a superior prognostic value when compared with current gold-standard clinical and molecular-based prognostication tools, and identifies novel molecular targets for precision treatment, representing a cutting-edge tool for predicting prognosis and guiding treatment across different stages of STS.

## Simple Summary

We assembled a novel cohort of over 100 high-grade soft tissue sarcoma (STS) samples and performed DNA sequencing (DNA-seq) and RNA sequencing (RNA-seq) to profile three of the most common STS subtypes. RNA-seq data was analyzed using unsupervised machine learning models, uncovering previously unknown molecular patterns and identifying four distinct transcriptomic subtypes with clear prognostic value (notable overall survival (OS) and disease-free survival (DFS) estimating capacity). Our transcriptomic subtype-based classification outperforms both SARCULATOR nomograms and CINSARC in terms of prognostic accuracy (superior OS predictive capability than SARCULATOR and CINSARC, and superior DFS predictive capability than CINSARC), being one of the first molecular-based classifications capable of predicting OS in STS. DNA-seq analysis revealed unique and previously unreported molecular targets across transcriptomic subtypes, highlighting potential opportunities for precision treatment. This new classification system represents a cutting-edge tool for predicting prognosis and guiding treatment across different stages of STS.

## 1. Introduction

Sarcomas are not modern vertebrate/human physiological defects or recently discovered pathological entities, but they have been almost invariably characterized as a group of rare and heterogenous mesenchymal malignancies [1,2,3,4]. Sarcomas’ heterogeneity is, conceptually, mainly a product of the currently applied histopathological classification system. This system fragments sarcomas into 50 to 150 histological subtypes, with approximately 20% of them being defined as “ultra-rare”, with an incidence of less than 1 in 1,000,000 [5].

Sarcomas histopathological classification system has important limitations. It is eminently morphological, and relies on the resemblance of neoplastic tissue to a type (line of differentiation) of normal tissue counterpart, being indirect and non-specific [6,7]. This system is intrinsically complex and error-prone, displaying overall diagnostic discrepancy rates of 28.2–56% and major diagnostic discrepancy (mainly due to discordances in histological types and grades) rates of 16.4–37% between referring and tertiary reference centers in different series [8,9,10,11,12,13].

This imperfect histopathological classification system impacts the management of soft tissue sarcomas (STSs), affecting both retroperitoneal (RPS) and extremity (eSTS) STS prognosis estimation and prognostication accuracy. Clinical nomograms, such as those in the SARCULATOR application, incorporate the STS histopathological subtype and other histopathological variables as critical factors for prognostication. Although sarcoma’s treatment is largely based on a “fit-for-all” principle, specific subtypes may require tailored approaches, meaning diagnostic inaccuracies can directly impact treatment decisions. In some series, histopathological reclassification altered treatment strategies in up to 15% of cases [13]. Furthermore, adding outstanding fragmentation and heterogeneity to sarcomas’ rarity creates a particularly deleterious context for pre-clinical studies and for early and late-phase clinical trials development (especially in what recruitment and design is concerned), hampering drug discovery and development in sarcomas. This negative influence on drug discovery and development also derives from this system’s static cytoarchitectural criteria, which do not fully capture the fluidity and dynamicity of the profusion of unique molecular landscapes of different sarcomas that are now being brought to light.

Molecular-based approaches, such as comprehensive genomic and transcriptomic profiling, may fill in some of the conceptual gaps of the histopathological classification system. For instance, whole-transcriptome sequencing reclassified 7% of STS histopathological diagnoses and identified treatment-relevant variants in 15% of STS cases in a particular study [14]. Other studies have reported diagnostic revision rates of 3–14% using genome-wide profiling [15,16,17]. These broad sequencing studies also found actionable molecular alterations in 31.7% of STS patients, while some real-world series identified druggable molecular alterations in 37.2% of STS patients, with 31.2% of them receiving personalized treatment based on the identified alterations [18].

Accordingly, various molecular-based approaches (single and multi-omics) are revealing new insights into sarcomagenesis, enabling a more granular and detailed mapping of pivotal sarcomas-defining molecular alterations, and are subsequently allowing for an increment of prognosis accuracy. Molecular prognostic and predictive biomarkers, such as genomic and transcriptomic signatures (e.g., CINSARC and CGI), along with proteomic and metabolomic fingerprints, are starting to pave the way for accurate prognosis definition and personalized treatment approaches identification in sarcomas [5,19,20,21,22].

The classification system should evolve from a crystalized and architectural archetype to a dynamic mesh that is capable of capturing and comprising both common molecular drivers and specific molecular adaptations, allowing researchers to better estimate prognosis (overcoming clinical-based nomograms such as SARCULATOR [23], gene expression-based signatures eminently related to mitosis and chromosome integrity, such as CINSARC [24], and even their combination, CINSARCULATOR [25]), and to better design studies and trials focused on tackling molecular alterations of sarcomas.

Here, we present a novel cohort of approximately 100 STS patients with rich genomic, transcriptomic and clinical characterization. We identified, from a multi-omics analysis, specific molecular signatures and transcriptomic subtypes that correlate with clinical outcomes, showing an independently validated prognostic value. Moreover, the prognostic value of gene expression signatures of particular STS transcriptomic subtypes benchmarks positively against CINSARC and SARCULATOR. In contrast to the current standard of care, the transcriptomic signature offers a reliable, non-subjective classification of STS. Altogether, this provides much-needed molecular-driven prognostication for STS, helping to guide selective personalized and precision treatment strategies with potential predictive utility.

## 2. Materials and Methods

A detailed description of the specific contribution of each of the participating institutions—Instituto de Medicina Molecular João Lobo Antunes (iMM), Instituto Português de Oncologia de Lisboa Francisco Gentil (IPOLFG), Instituto Superior Técnico (IST), F.Hoffmann-LaRoche and Foundation Medicine—may be found in Author Information-Contributions (see Contributions).

Ethical considerations are also provided in Ethics Declarations (see Ethics Declarations).

### 2.1. Sample Characterization

This study has included 102 formalin-fixed paraffin-embedded (FFPE) neoplastic tissue samples from 101 STS patients diagnosed and treated at IPOLFG (a tertiary oncological center, one of the sarcoma European reference centers) between 15 April 2013 and 29 September 2022.

These samples were previously stored at the IPOLFG tumor biobank, and were part of this biobank sarcoma collection.

The sample pool comprised 26 dedifferentiated liposarcoma (DDLPS) samples, 25 high-grade leiomyosarcoma (LMS) samples, and 51 undifferentiated pleomorphic sarcoma (UPS) samples.

A sarcoma-dedicated pathologist reviewed each of the 102 STS samples. The pathologist scored the images for all the 102 samples that were shipped to Foundation Medicine, Inc. (Cambridge, MA, USA) for molecular profiling. The number of slides available for review from each case ranged from 1 to 6. Pathology reports were reviewed for sarcoma site, depth, FNCLCC grade, presence of multifocality, completeness of resection, reported immunohistochemical studies and/or molecular diagnostics and, subsequently, histopathological diagnoses.

The research team analyzed clinical files, retrieving data not only from IPOLFG institutional records, but also from accessible national electronic clinical files. An anonymized database has been developed specifically for this study. This database includes detailed information on patient demographics, sarcomas’ characteristics, treatment strategies (both neoadjuvant and adjuvant), surgical data, and oncological follow-up. The most recent follow-up has been conducted on 10 October 2023.

### 2.2. Samples Circuit

A formal histopathological review was firstly conducted at IPOLFG. The FFPE blocks were then transported to iMM where they were sectioned by the Comparative Pathology Unit team. The slides obtained were stored at the Translational Oncobiology Laboratory at iMM, while the blocks were shipped to Foundation Medicine, Inc. The samples were shipped in three different batches—the first one, including 26 samples of DDLPS, was shipped on July 2022; the second one, including 25 samples of LMS, was shipped on October 2022; the third one, including 51 samples of UPS, was shipped on January 2023.

### 2.3. DNA and RNA Sequencing

A total of 102 FFPE STS samples from 101 patients were characterized using FoundationOne^®^CDx (F1CDx^®^) for DNA sequencing (DNA-seq) and FoundationOne^®^RNA (F1RNA) for RNA sequencing (RNA-seq). Testing was performed in a Clinical Laboratory Improvement Amendments (CLIAs)-certified, College of American Pathologists (CAPs)-accredited, New York State-approved laboratory (Foundation Medicine, Inc., Cambridge, MA, USA). DNA and RNA were simultaneously co-extracted and isolated from the FFPE samples. F1CDx is a next generation sequencing (NGS)-based assay for the detection of short variants (substitutions and short insertions/deletions [indels]), copy number alterations (CNAs), and large genomic rearrangements in 324 cancer-associated genes, as well as reporting of complex biomarkers including microsatellite instability (MSI) and tumor mutational burden (TMB). The clinical and analytical validation for F1CDx has been published by Milbury et al. [26]. F1RNA is a laboratory developed test that uses hybrid-capture-based targeted RNA-seq designed for optimal detection of cancer-related gene fusions and rearrangements for 318 genes for clinical use and gene expression profiling (GEP) for 1517 genes for research use only (RUO). Analytical validation studies for fusion detection have been previously performed to assess fusion calling accuracy, reproducibility, and limit of detection in 189 clinical solid tumor specimens [27]. The results from both DNA-seq and RNA-seq were periodically sent back to iMM, IPOLFG, and IST via an encrypted and safe platform.

### 2.4. DNA-seq and RNA-seq Data Analysis

#### Population Considered for Molecular Analysis

Out of the 102 STS samples sent for molecular analysis, 79 samples passed F1CDx quality control and were sufficient for DNA analysis, and 75 samples passed F1RNA quality control and were sufficient for RNA-seq expression analysis. One additional sample was excluded from the RNA-seq expression analysis after being identified as an outlier using the principal component analysis (PCA). A total of 74 samples (16 DDLPS, 15 LMS and 43 UPS) were therefore considered for downstream expression analysis.

Of note, 53 of the 75 samples were excluded for analysis of fusions in RNA due to not passing the post-sequencing QC metrics required for clinical RNA rearrangement detection.

The disparity between these QC passage rates for the optimal detection of cancer-related gene fusions and rearrangements (318 gene panel) test and the gene expression profiling (1517 gene panel) test covered by FoundationOne^®^RNA lies on the different stringencies of each of these tests, considering that one has been developed for clinical use and the other has been designed for research use only.

### 2.5. RNA-seq Expression Data Analysis—Transcriptomic Clusters Discovery

The computational analyses were performed using R (v4.4.0). RNA-seq data from these 74 samples was filtered for expression using the edgeR (v4.2.1) [28] filterByExpression method to remove lowly expressed genes, followed by Voom normalization to stabilize variance across samples. The genes were then filtered based on Mean Absolute Deviation, retaining the top 55% of the most variable genes. Consensus clustering from the ConsensusClusterPlus package (v1.68.0) [29] was applied and evaluated using the Elbow method, which identified a statistically optimal number of 4 clusters (Supplementary Figure S1b). Differential gene expression analysis was then conducted through pairwise comparisons between clusters using the Limma package (v3.60.4) [30] and *p*-values were adjusted for multiple hypothesis testing using the Benjamini–Hochberg False Discovery Rate (BH-FDR). The unique genes defining each cluster were identified by intersecting the genes that were differentially expressed in the same direction within a specific cluster resulting in the gene sets C1_under, C1_over, C2_under, C2_over, C3_under, C3_over, C4_under, C4_over.

### 2.6. DNA-seq Genomic Alteration Data Analysis—Genomic Alterations/Variants (Found in the Patients Included in Each Transcriptomic Cluster) Actionability Evaluation

We extracted the alterations detected by F1CDx in the patients included in each of the 4 transcriptomic clusters. To systematically analyze the actionability of each of the alterations highlighted, the Karolinska Molecular Tumor Board Portal (MTBP) [31] was used. MTBP offers a general framework for the interpretation of the functional and predictive value of a given list of cancer genomic variants by using several computational tools and databases that are referenced in the provided results.

### 2.7. Transcriptomic Clusters/Subtypes Clinical Significance Assessment

#### 2.7.1. Evaluation of Transcriptomic Clusters/Subtypes Intrinsic Prognostic Value (Study Cohort)

Overall Survival (OS) was defined as the time from the date of surgery to the date of the last recorded clinical observation or date of death from any cause. Disease-Free Survival (DFS) was defined as the time from the date of surgery to the date of detection of local or distant recurrence, or date of death, and, for patients without any of these events, to the date of last recorded clinical observation. Follow-up time was calculated from the date of surgery until the date of the last recorded clinical observation (for living patients) or date of death (for dead patients).

OS and DFS analyses were performed using the survival package (v3.7-0) [32]. These analyses only contemplated patients whose samples were considered for the RNA-seq expression analysis. A total of 74 patients (corresponding to 74 samples) were integrated. Out of these 74 patients, 4 had not been submitted to a surgical approach and were, therefore, excluded. In total, 70 patients were considered for this analysis.

Each sample was accordingly classified using consensus clustering as described in “RNA-seq data analysis—Transcriptomic clusters discovery”. The distribution of these samples per transcriptomic cluster was considered for OS and DFS estimation. Cox Proportional Hazards Models for OS and DFS were then applied, also incorporating other relevant clinical variables. The Schoenfeld residuals were used to validate each variable, ensuring time independence. As a result, distant metastasis was excluded from the OS model due to its violation of this assumption. An Analysis of Variance (ANOVA) test was subsequently performed on the Cox Proportional Hazards Model.

The TCGA-SARC dataset was used as a validation dataset [33]. This dataset was first filtered for specific subtypes (DDLPS, LMS, UPS) (*n* = 127). It was processed similarly to our dataset, i.e., normalized using the edgeR (v4.2.1) filterByExpression method followed by Voom quantile normalization from the Limma package. After normalization, the single-sample gene set enrichment analysis was performed using the Corto package (v1.2.4) [34], with KEGG pathways (*n* = 186) and the gene sets corresponding to under- and over-expressed genes in each cluster (C1_under, C1_over, C2_under, C2_over, C3_under, C3_over, C4_under, C4_over) passed as inputs. The *p*-values for each pathway were normalized using BH-FDR and KEGG pathways were subsequently filtered out after adjusting *p*-values across the entire set of pathways. By considering KEGG pathways in our enrichment analysis, we were able to assess the enrichment of each of the transcriptomic clusters in terms of specific pathways and compare it with other biological processes. Finally, each TCGA-SARC sample was classified based on the gene set with the most significant adjusted *p*-value (*p* < 0.05). Similar to previous OS and DFS analyses, the Kaplan–Meier log-rank tests for OS and DFS were then applied to compare the survival outcomes across clusters, and the Cox Proportional Hazards Models for OS and DFS were then again employed, incorporating relevant clinical variables.

The process of excluding a potential contamination effect by the preponderance of UPS samples in the global pool of samples used for this analysis and in the composition of the majority of the transcriptomic clusters, was mainly focused on the verification of persistence of patterns of molecular enrichment and of the prognostic value of transcriptomic clusters/subtypes.

Considering that the most commonly represented STS histopathological subtype in 3 of the 4 identified transcriptomic subtypes is UPS and the pronounced relative preponderance of UPS in transcriptomic subtypes 3 and 4, a potential “contamination” effect by UPS samples and the notion that these 4 subtypes could be portraying solely the UPS molecular landscape and respective intrinsic subtypes had to be ruled out. Focusing our attention on the study cohort, the removal of the UPS samples would lead to a number of remaining samples (DDLPS and LMS) that would be too low to allow for the performance of an unsupervised consensus clustering analysis. Therefore, using the TCGA-SARC dataset, we evaluated if the removal of UPS patients from the patients pool would alter the previously verified molecular enrichment of these STS patients’ samples in the transcriptomic subtypes and would modify any statistically significant correlation that had been previously verified between the transcriptomic clusters-based classification and OS.

#### 2.7.2. Evaluation of Transcriptomic Clusters/Subtypes Relative and Comparative Prognostic Value (External Cohorts)

Patients of the study cohort were classified using the specific clinical nomograms (either for RPS and eSTS) available at SARCULATOR (https://www.sarculator.com/, accessed on 15 October 2024), to estimate the 5-year survival probability for each patient. Then, they were stratified according to SARCULATOR’s predefined prognostic groups (5-year OS > 60% vs. 5-year OS ≤ 60%). Out of the 70 patients whose samples were considered for the RNA-seq analysis and that were submitted to a surgical approach, 67 were successfully classified using the up mentioned nomograms. The patients for whom the nomograms could not be applied were not classified either due to the lack of crucial data necessary to use the nomograms or due to the presence of tumor fragmentation, which prevented accurate estimation of tumor size. Various C-index values were then compared, derived from the Cox Proportional Hazards Model for OS. The comparisons included the following models: SARCULATOR 5-year OS prediction; transcriptomic clusters; SARCULATOR 5-year OS prediction combined with transcriptomic clusters; and finally, transcriptomic clusters combined with age. Taking the SARCULATOR stratified patients (5-year OS > 60% vs. 5-year OS ≤ 60%), Kaplan–Meier curves were generated for each of the transcriptomic clusters and a comparative analysis was then performed.

We also performed the same analysis using an external cohort, namely, the TCGA-SARC cohort. We classified the patients integrated in the TCGA-SARC cohort with SARCULATOR and consequently obtained a 5-year OS probability for each patient. Then, we calculated the C-Indexes (derived from different Cox Proportional Hazards Models for OS) and compared them. We considered different models and distinct model combinations, including SARCULATOR and TC.

CINSARC classification was applied to the TCGA-SARC dataset, based on a previous study that had already classified TCGA-SARC data using CINSARC [35]. Using the available CINSARC code (https://codeocean.com/capsule/4933686/tree/v1, accessed on 15 October 2024), TCGA subtypes (DDLPS, UPS, and LMS) were reclassified according to the CINSARC C1 and C2 categories. Kaplan–Meier curves were then generated to evaluate OS predictions based on CINSARC classification and transcriptomic clusters. Additionally, a Cox Proportional Hazards Model was employed to assess OS, also incorporating other relevant clinical variables for a more comprehensive analysis. Finally, we incorporated the CINSARC gene list in the ssGSEA cluster assignment and analyzed the normalized enrichment scores (NESs), using Spearman’s rank test to correlate the enrichment scores of each gene set.

As previously described, we performed a comparative analysis, using the TCGA-SARC cohort, of the C-Indexes of different survival estimation models, including SARCULATOR, CINSARC, and transcriptomic clusters.

## 3. Results

### 3.1. Clinical Characteristics of the Study Cohort

The 102 samples that were used for this study were obtained from 101 patients. These 101 patients displayed a median age of 67 (IQR 19.7) years old, and a balanced gender distribution (50.5% male) (Supplementary Table S1). Twenty-five (25) patients had a diagnosis of dedifferentiated liposarcoma (DDLPS), 25 patients had a diagnosis of leiomyosarcoma (LMS), and 51 patients had a diagnosis of undifferentiated pleomorphic sarcoma (UPS). These patients STS’s were predominantly located in the lower limb (*n* = 49, 48.5%), followed by the retroperitoneum (*n* = 31, 30.5%). The primary malignant tumors (sarcomas) had a median size of 13 (IQR 10.0) cm. Two (6.5%) of the 31 retroperitoneal sarcomas were multifocal. All of these 101 patients’ samples were of high-grade (Grade 3). The great majority of cases (*n* = 96, 95%) presented with localized disease. Five (5%) patients were metastatic at diagnosis. Among these five patients, four (80%) had lung metastases and one (20%) had ganglionic mediastinal metastases. Three of these five patients (60%) were submitted to surgery with a palliative intent and two (40%) were not surgically interventioned.

Surgery was the most frequently employed treatment strategy (*n* = 99, 98.0%). Among the 99 patients that were surgically interventioned, two (2%) had already been operated in another institution. From the 97 patients that were submitted to surgery by the IPOLFG surgical team, 94 (96.9%) were operated with a curative intent and three (3.1%) with a palliative intent. Among the 94 patients that were operated with curative intent, two (2.1%) had sarcomas that were deemed unresectable during surgery and 92 (97.9%) had resectable disease (Supplementary Table S2). The resection margin status was R0/R1 in 96.7% (*n* = 89) of cases. Among the 94 patients that were operated with a curative intent, three (3.2%) were submitted to neoadjuvant treatment, two were treated with neoadjuvant chemotherapy (a doxorubicin-ifosfamide-based regimen was used in both cases), and one with neoadjuvant external radiotherapy (50 Gy/25 fractions). Fifty-eight (63.0%) patients received adjuvant treatment, primarily external radiotherapy (*n* = 55, 94.8%).

Three patients with an R2 resection were excluded from the pool of locally recurrent cases since they were considered to have persistent disease. Different oncological outcomes were evaluated for the patients who were submitted to a resection with curative intent (*n* = 94), and whose resection margins were R0/R1 (*n* = 89, 94.7%), during a median follow-up period of 27 (IQR 51.3) months since their diagnosis. Of these 89 patients, 29 (32.6%) had already had a previous local treatment in another institution (surgery or radiotherapy). Among these 89 patients, the local recurrence rate was 46.1% (*n* = 41), with a median time to local recurrence of 14 (IQR 29.0) months. Among the 96 patients without distant metastasis ab initio, the distant metastasis rate was 41.7% (*n* = 40). Metastases were mostly found in the lungs (*n* = 34, 85%), with a median time to distant metastasis of 13 (IQR 17.2) months. The metastasis-free survival (MFS) rate during the follow-up period was 34.4% (*n* = 33) and the OS rate during the follow-up period for these patients was 43.8% (*n*= 42) with a median follow-up of 27 (IQR 51.5) months. The 5-year MFS rate and the 5-year OS rate for these patients were 37% and 46%, respectively. When all the patients are considered (*n* = 101), the OS rate during the follow-up period was 42.6% (*n* = 43), with a median follow-up of 25 (IQR 51.9) months. The 5-year OS rate for all the 101 patients was 44%.

### 3.2. Unsupervised Machine Learning Identifies Four Transcriptomic Subtypes

Data from 74 samples (16 DDLPS, 15 LMS, and 43 UPS) of 74 patients was considered for the RNA sequencing (RNA-seq) analysis (see Section 2).

**Figure 1:**
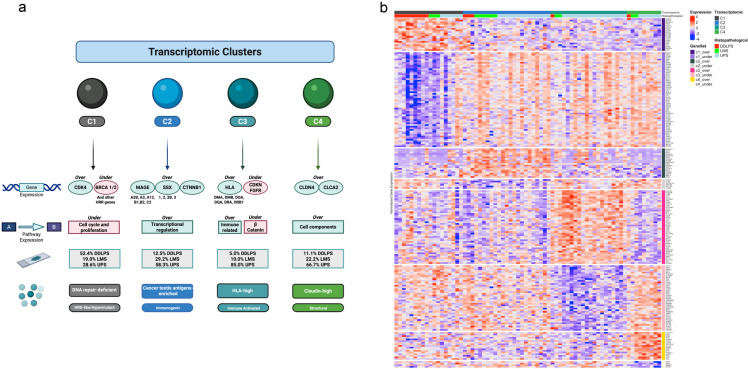
The four identified transcriptomic clusters and their defining molecular features. (**a**) Schematic of the genes and pathways whose expression pattern most distinctively portrays each cluster and of the histopathological subtypes that differentially compose each cluster. Created in BioRender. Esperança-Martins, M. (2025) https://BioRender.com/c93w707 (accessed on 18 August 2025). (**b**) Heatmap plot displaying clinical and molecular (normalized gene expression data) features of each transcriptomic cluster.

Transcriptomics consensus clustering identified four transcriptomic clusters (the optimal number of clusters was found using the Elbow method) (Supplementary Figure S1a,b). Each transcriptomic cluster is portrayed by differential expression, either over or under expression, of a certain plethora of genes and of associated pathways (Figure 1a,b, Supplementary Figure S2 and Table S1).

For each cluster, the differential gene expression analysis and subsequential KEGG pathway enrichment analysis were used to portray the cluster individual molecular landscape.

Cluster 1 (C1) is more distinctively portrayed by the over expression of genes that encode cyclin-dependent kinases and cyclins such as *CDK4* and *CCND2*. This cluster is also characterized by the over expression of genes that encode chemokines and transcription factors and by the under expression of an impressive array of genes that are involved in DNA homologous recombination repair (HRR) mechanisms, such as *BRCA1*, *BRCA2*, *FANCD2*, *PALB2*, *RAD51*, *CHEK1*, and *BRIP1*. Globally, there is an under expression of cell cycle and proliferation pathways (probably associated with the under expression of a significant number of genes involved in HRR and of a number of genes encoding cyclins, other than CCND2, as shown in Table A1). C1 is the DNA repair-deficient (Homologous recombination deficient (HRD)-like/Hypermutant) cluster, defined by the under expression of HRR genes. This cluster is mainly composed of samples that were classified, according to the currently used histopathological classification, as DDLPS (52.4%), with UPS (28.6%) and LMS (19.0%) samples also being integrated in this cluster.

Cluster 2 (C2) is predominantly defined by the over expression of different cancer testis antigens (CTAs) genes, namely, a plethora of MAGE genes (such as *MAGEA2B*, *MAGEA3*, *MAGEA12*, *MAGEB1*, *MAGEB2* and *MAGEC2*), and different SSX genes (such as *SSX1*, *SSX2*, *SSX2B* and *SSX3*). The over expression of *CTNNB1* is also verified in this cluster. C2 is the cancer testis antigens-enriched (Immunogenic) cluster, characterized by a strong expression of MAGE and SSX genes and by the over expression of transcription regulation pathways. It is important to emphasize that our study cohort included DDLPS, LMS and UPS samples, not comprising either synovial sarcoma or myxoid/round cell liposarcoma samples. There is an over expression of transcriptional regulation pathways in this cluster. This cluster is mostly composed of samples classified as UPS (58.3%), with LMS (29.2%) and DDLPS (12.5%) samples also being represented in this specific cluster.

Cluster 3 (C3) is specifically characterized by the over expression of genes that encode Major Histocompatibility Complex (MHC) class II/Human Leukocyte Antigen (HLA) class II (*HLA-DMA*, *HLA-DMB*, *HLA-DOA*, *HLA-DQA*, *HLA-DRA* and *HLA-DRB1*) genes. Besides HLA class II genes, an over expression of *TGFβ1*, *ETV5*, *BTK* and *BATF* genes is also verified. On the other hand, the under expression of CDKN (*CDKN1C* and *CDKN2A*) and FGFR (*FGFR2* and *FGFR3*) genes also characterizes this cluster. In terms of pathways, this cluster is marked by an over expression of immune-related pathways and an under expression of the β-catenin pathway. C3 is the HLA-high (Immune activated) cluster, portrayed by the over expression of HLA class II genes, and by an enrichment in immune pathways expression. Samples labeled as UPS (85.0%) are predominant, while LMS (10.0%) and DDLPS (5.0%) samples are also integrated in this cluster.

Cluster 4 (C4) is represented by the over expression of a plethora of genes that encode different structural protein elements, such as claudin (*CLDN*) 4 (this gene encodes a membrane protein that is a component of epithelial cell tight junctions), *CLCA2*, and *GAS7*. In addition, there is an over expression of other genes such as *SMAD3* and *PDGFD*. Interestingly, an under expression of *ACTN1* is verified. There is an overall over expression of cell components pathways. C4 is the claudin-high (Structural) cluster, characterized by the over expression of genes encoding claudin and other cell adhesion/structural proteins. This cluster is also principally composed of UPS (66.7%) samples, incorporating both LMS (22.2%) and DDLPS (11.1%) samples.

Each transcriptomic cluster defines and corresponds to a transcriptomic subtype.

### 3.3. Independent Validation of the Prognostic Value (In Terms of OS and DFS) of the Identified Transcriptomic Subtypes

**Figure 2:**
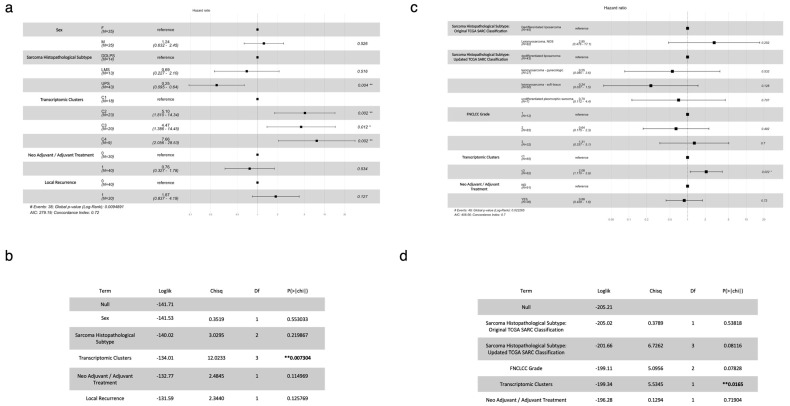
Transcriptomic clusters/subtypes and their respective molecular signatures exquisite prognostic value, as assessed by Cox Proportional Hazards Models with hazard ratios and 95% confidence intervals, and ANOVA tests. (**a**) Forest plot showing the results of the evaluation of the differential impact of distinct demographical, clinical, histopathological and molecular variables on OS in the study cohort using a Cox Proportional Hazards Model. (**b**) Table displaying the results of the ANOVA test applied to the Cox Proportional Hazards Model to assess the predictive ability of different variables for OS estimation in the study cohort. (**c**) Forest plot showing the results of the evaluation of the differential impact of distinct histopathological and molecular variables on OS considering the TCGA-SARC patients (classified in accordance with the transcriptomic clusters-based classification) using a Cox Proportional Hazards Model. (**d**) Table displaying the results of the ANOVA test applied to the Cox Proportional Hazards Model to assess the predictive ability of different variables for OS estimation in the validation cohort (TCGA-SARC). ** *p*-value < 0.01; * *p*-value <0.05 and >0.01; # Number of.

The newly identified transcriptomic clusters/subtypes were included, alongside other key demographical, clinical, and histopathological data in the pool of variables that were considered for analysis using a Cox Proportional Hazards Model to estimate and compare the differential impact of each of these variables on OS (using the study cohort). This analysis revealed that subtypes C2, C3, and C4 are negative prognostic factors. Specifically, the hazard ratios (HRs) that were found were C2 (HR 5.10; 95% CI 1.81–14.34; *p* = 0.002), C3 (HR 4.47; 95% CI 1.39–14.45; *p* = 0.01), and C4 (HR 7.66; 95% CI 2.06–28.53; *p* = 0.002) (Figure 2a). An Analysis of Variance (ANOVA) test was applied to the Cox Proportional Hazards Model and demonstrated that transcriptomic clusters/subtypes were the variable with the most significant correlation with OS (*p* < 0.01) (Figure 2b). The inclusion of other variables, such as age and treatment modality, in a similar analysis employing a Cox Proportional Hazards Model did not modify the negative prognostic impact of C2, C3 and C4, which remained significant (Supplementary Figure S3). The respective ANOVA test confirms, once again, transcriptomic clusters/subtypes as the variable with the most significant correlation with OS (*p* < 0.01).

To externally validate these findings, we used the TCGA-SARC dataset, namely, the data of patients with the same STS histopathological subtypes as the patients included in our study cohort (DDLPS, LMS and UPS) (*n* = 127). We employed normalized gene expression data to reclassify patients into our transcriptomic cluster-specific gene signatures using single-sample Gene Set Enrichment Analysis (ssGSEA) (see Section 2). Patients were assigned to the transcriptomic subtype with the lowest significant FDR-adjusted p-value. This led to the classification of these TCGA-SARC patients either into C1 (*n* = 65) or C3 (*n* = 62). Accordingly, a significant enrichment of the TCGA-SARC patients’ samples to C1_under and C3_over was verified (Supplementary Figure S4). An analysis using a Cox Proportional Hazards Model was carried out, incorporating the histopathological classification that was originally used in TCGA-SARC (that grouped LMS and UPS together), a recently proposed histopathological classification that distinguishes gynecological LMS, soft tissue LMS and UPS (and which is currently used for patient stratification), the FNCLCC grade, the transcriptomic clusters/subtypes, and the use of neoadjuvant/adjuvant treatment. This analysis confirmed that C3-enriched patients have a worse prognosis (HR 2.08; 95% CI 1.11–3.9; *p* = 0.022) (Figure 2c). An ANOVA test of the Cox Proportional Hazards Model showed, once again, that the transcriptomic cluster-based classification was the most significant predictor of OS (*p* = 0.0165) (Figure 2d). For censored patients (alive at the last follow-up date), the median follow-up was 37.8 months (IQR: 13.0–64.0; range: 2.8–97.2 months) in our study cohort and 37.2 months (IQR: 26.6–63.0; range: 0.5–171.0 months) in the TCGA-SARC cohort. The similarity in median and mean follow-up times across cohorts supports the robustness and comparability of the results.

We performed similar analyses to estimate and compare the differential impact of transcriptomic clusters/subtypes and the same array of additional variables on disease-free survival (DFS) for the study cohort and, afterwards, for the TCGA-SARC cohort. A Cox Proportional Hazards Model-based analysis revealed that subtypes C2 (HR 3.69; 95% CI 1.33–10.20; *p* = 0.012) and C3 (HR 3.68; 95% CI 1.15–11.77; *p* = 0.028) are negative prognostic factors and that neoadjuvant/adjuvant treatment is a positive prognostic factor (HR 0.32; 95% CI 0.14–0.74; *p* < 0.01) in the study cohort (Supplementary Figure S5a). Once again, the inclusion of other variables, such age and treatment modality, did not influence or modify the negative prognostic impact of C2 and C3, which remained significant (Supplementary Figure S5b). An ANOVA test was applied to this Cox Proportional Hazards Model, and demonstrated that transcriptomic clusters/subtypes were, alongside neoadjuvant/adjuvant treatment (as expected) (*p* = 0.012), a variable with a significant correlation with DFS (*p* = 0.042) (Supplementary Figure S5c). We used, once again, the TCGA-SARC dataset to externally validate these findings and proceeded as explained above for OS. The Cox Proportional Hazards Model-based analysis confirmed that C3-enriched patients have a worse prognostic profile, even though its impact on DFS is not statistically significant (HR 1.43; 95% CI 0.96–2.1; *p* = 0.078), while neoadjuvant/adjuvant treatment has, in this cohort, a negative prognostic impact, even though its impact on DFS is also not statistically significant (HR 1.19; 95% CI 0.70–2.0; *p* = 0.510) (Supplementary Figure S5d). The ANOVA test that was applied to this Cox Proportional Hazards Model demonstrated that transcriptomic clusters/subtypes were the most significant predictors of DFS (*p* = 0.041) (Supplementary Figure S5e). Moreover, a DFS analysis by the Kaplan–Meier log-rank test was performed for patients of this TCGA-SARC cohort, classified per transcriptomic cluster/subtype (C1 and C3), and showed statistically significant differences, with C3 displaying a worse DFS (log rank *p* = 0.043) than C1. This analysis shows that our transcriptomic clusters/subtypes also capture STS populations with different DFS profiles (Supplementary Figure S6).

### 3.4. Enrichment of Transcriptomic Subtypes C1 and C3 in TCGA-SARC and Survival Correlation Is Independent from UPS

As described in the Methods section, using the TCGA-SARC dataset, we evaluated if the removal of UPS patients from the patients’ pool would alter the previously verified molecular enrichment of these STS patients’ samples in C1 and C3 (C1_under; C3_over) and modify the previously verified statistically significant correlation between the transcriptomic clusters-based classification and OS. When UPS patients were removed from the considered TCGA-SARC patients’ population, the molecular enrichment of the population in C1 and C3 (C1_under; C3_over) (Supplementary Figure S7) and the correlation between the transcriptomic clusters-based classification and OS remained statistically significant (namely, the correlation between C3 and OS) (Supplementary Figure S8).

### 3.5. Molecular Signature/Transcriptomic Cluster-Based Classification Outperforms SARCULATOR in Terms of Prognostic Value

We conducted a comparative analysis between the prognostic values of a molecular signature/transcriptomic cluster-based classification and the clinical nomograms available at SARCULATOR (SARCULATOR). A total of 67 patients within the study cohort had their 5-year OS probability estimated following the use of SARCULATOR nomograms (see Section 2). The median 5-year predicted OS was 57% (IQR 26.5%).

C-indexes of the different Cox Proportional Hazard Models for OS were calculated and then compared (see Section 2). The following models were considered: SARCULATOR 5-year OS prediction (SARC); transcriptomic clusters (TCs); SARCULATOR 5-year OS prediction combined with transcriptomic clusters (SARC + TCs); and finally, transcriptomic clusters combined with age (TCs + AGE).

**Figure 3:**
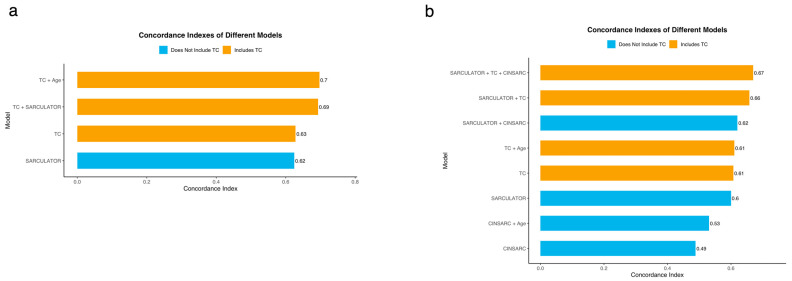
The transcriptomic cluster-based classification outperforms the SARCULATOR clinical nomograms in terms of prognostic value (OS), as assessed by concordance index (C-Index) comparisons. (**a**) Bar chart displaying the concordance indexes of different prognostic models employed using the population of the study cohort (including SARC, TC, TC + SARC and TC + Age). (**b**) Bar chart showing the concordance indexes of different prognostic models employed using the population of the validation cohort (TCGA-SARC) (including CINSARC, CINSARC + Age, SARC, TC, TC + Age, SARC + CINSARC, SARC + TC, SARC + TC + CINSARC).

The TC + AGE model showed the strongest OS predictive ability and the best prognostic value (C-index 0.7, Figure 3a). Notably, the transcriptomic cluster-based classification outperformed the SARCULATOR nomograms in terms of prognostic value (C-index of 0.63 vs. 0.62, respectively). This suggests that, even without the incorporation of age or without being particularly designed or trained to specifically predict OS, the transcriptomic cluster-based model offers superior prognostic accuracy than the SARCULATOR nomograms (which include age as a necessary variable for its calculation). Furthermore, the TC + AGE model showed a clearly superior prognostic value than the SARC model (C-index of 0.7 vs. 0.62, respectively), which is also noteworthy.

Altogether, these results, in an analysis within our study cohort, point towards a superior prognostic value of the transcriptomic cluster-based classification over the currently employed gold-standard clinical nomograms approach.

### 3.6. Independent Validation of the Enhanced Prognostic Value of the Molecular Signature/Transcriptomic Cluster-Based Classification

We sought to validate the superior prognostic value when compared with clinical-based prognostication tools, of the transcriptomic cluster-based classification using an independent/external cohort. We performed the same analysis for the TCGA-SARC cohort [33] by calculating the C-Indexes of different Cox Proportional Hazard Models for OS and comparing them. Distinct model combinations were considered, including SARCULATOR, CINSARC (classification of the patients of the TCGA-SARC cohort using CINSARC was possible, in contrast to what was verified for the patients of the study cohort as reported in Results-A transcriptomic cluster-based classification outperforms the CINSARC expression-based signature in terms of prognostic value and Methods) and TC as features.

In this cohort, the results of our analysis reinforced our findings, with TC outperforming, albeit marginally, SARCULATOR (C-Index of 0.61 vs. 0.6, respectively) (Figure 3b). Notably, the addition of age to the TC model did not affect its performance, with TC + AGE showing the same C-Index of 0.61 as TC.

Additionally, TC (C-Index of 0.61) consistently outperformed CINSARC (C-Index of 0.49) and CINSARC + AGE (C-Index of 0.53), demonstrating that our molecular-based classification displays a superior prognostic value than the currently used molecular-based classification.

Importantly, the best-performing models were those incorporating TC. The combination of TC with SARCULATOR achieved a C-Index of 0.66, while the combination of TC with SARCULATOR and CINSARC further improved performance, achieving the highest C-Index of 0.67. These findings validate the robustness of the transcriptomic cluster-based classification and highlight its critical role in enhancing the accuracy of prognostic models when combined with clinical and molecular predictors.

Although neither SARCULATOR (which displays OS predictive capacity), nor CINSARC (which has metastasis-free survival (MFS) predicting ability) are provenly specific predictors of DFS, we also performed a similar analysis for the TCGA-SARC cohort [33], by calculating the C-Indexes of different Cox Proportional Hazard Models for DFS and comparing them. The model combinations that were used for the OS analysis were also used for this DFS analysis.

The results of these analyses show that SARCULATOR marginally outperforms TC (C-Index 0.57 vs. 0.56, respectively), while TC outperforms CINSARC (C-Index 0.56 vs. 0.52) (Supplementary Figure S9). However, once again, the best performing models were the ones that incorporated TC, with its combination with SARCULATOR reaching the highest C-Index (0.59) and its combination with age equaling SARCULATOR’s isolated C-Index (0.57). Indeed, TC may also have a relevant relative prognostic value in terms of DFS among other clinical and molecular-based prognostication tools, especially if combined with clinical-based prognostication tools or isolated clinical features.

### 3.7. Molecular Signature/Transcriptomic Cluster-Based Classification Enable Prognostic Sub-Stratification Within SARCULATOR-Defined Prognostic Groups

Additionally, we attempted to understand if the application of our transcriptomic cluster-based strategy could identify and sub-stratify patients with different prognostic horizons inside the same SARCULATOR-defined prognostic groups (predicted 5-year OS > 60% vs. predicted 5-year OS ≤ 60%).

Sub-stratification inside the favorable prognostic group, defined by patients with a predicted 5-year OS > 60%, could spot patients with a distinct prognostic profile according to the transcriptomic cluster/subtype their STS belongs to and that, in the case of a relative negative prognostic profile, might benefit from an early and tailored adjuvant systemic treatment approach and/or a more intensive surveillance approach. Sub-stratification inside the unfavorable prognostic group, defined by patients with a predicted 5-year OS ≤ 60%, could identify, among the pool of patients that collectively display an indication for adjuvant chemotherapy, patients with worse relative prognosis, whose adjuvant systemic treatment approach should potentially be intensified (either in terms of number or doses of systemic treatment agents, or in terms of frequency of treatment cycles) and patients with a better prognosis, whose adjuvant treatment approach could be, relatively, less aggressive (also in the same terms that have been previously mentioned, but in the opposite direction).

Among the 67 patients of the study cohort whose classification with SARCULATOR was amenable to be performed, 30 displayed a predicted 5-year OS > 60% and 37 showed a predicted 5-year OS ≤ 60%.

By performing a survival analysis using the Kaplan–Meier method, the transcriptomic clusters-based classification was able to significantly sub-stratify patients with different prognostic horizons within the unfavorable prognostic group (predicted 5-year OS ≤ 60%) (*p*-value 0.018), while it was not able to significantly sub-stratify patients with distinct prognostic profiles within the favorable prognostic group (predicted 5-year OS > 60%) (*p*-value 0.78) (Supplementary Figure S10). Considering the unfavorable prognostic group, there is a statistically significant difference in OS between patients whose STS belongs to C1 and patients whose STS belongs to non-C1 subtypes (C2, C3 and C4). Patients whose STS belongs to C1 display a better relative prognosis.

Next, we performed an identical survival analysis in the TCGA-SARC cohort. The transcriptomic clusters-based classification was not able to significantly sub-stratify patients with distinct prognostic profiles either within the favorable prognostic group (predicted 5-year OS > 60%) (*p*-value 0.14) or within the unfavorable prognostic group (predicted 5-year OS ≤ 60%) (*p*-value 0.28) (Supplementary Figure S11a).

If only patients from the TCGA-SARC cohort with a grade 3 DDLPS, LMS and UPS were considered, the transcriptomic clusters-based classification would still not be able to significantly sub-stratify patients with distinct prognostic profiles either within the favorable prognostic group (predicted 5-year OS > 60%) (*p*-value 0.32) or within the unfavorable prognostic group (predicted 5-year OS ≤ 60%) (*p*-value 0.6) (Supplementary Figure S11b). However, it is important to note that, even lacking statistical significance, the survival curves indicate the tendency of a better prognosis for patients with a C1 STS, when compared with patients with a non-C1 STS. This apparently discordant finding (within the study cohort and within the validation cohort) may be explained by some statistical data regarding the study populations: among the 127 patients included in the TCGA-SARC that have a formal diagnosis of DDLPS, LMS and UPS, only 33 have a grade 3 STS. Of these 33 patients with a grade 3 DDLPS, LMS or UPS, only 23 display a predicted 5-year OS ≤ 60% (estimated using SARCULATOR). Our study cohort includes a higher absolute number (*n* = 37 vs. *n* = 23) and a higher proportion (37/70; 52.9% vs. 23/127; 18.1%) of patients with a grade 3 STS that display a predicted 5-year OS ≤ 60%.

### 3.8. Molecular Signature/Transcriptomic Cluster-Based Classification Outperforms CINSARC in Terms of OS Predictive Capacity

We also compared the prognostic values of the transcriptomic cluster-based classification with the Complexity INdex in SARComas (CINSARC) (an expression-based signature related to mitosis and chromosome integrity), using patients from the TCGA-SARC dataset with the same STS histopathological subtypes as the patients that were included in our study cohort (DDLPS, LMS and UPS) (*n* = 127).

CINSARC annotation of our study cohort was not possible since the FoundationOne^®^RNA gene set does not include 32 of the genes included in CINSARC (48% of the total number of genes considered in CINSARC) (listed in detail in Supplementary Materials). CINSARC annotation of a cohort requires that all of the 67 genes that compose this molecular signature are covered by the gene set of the sequencing test that is employed to map the transcriptomic landscape of that cohort. This way, comparisons between the accuracy power of the transcriptomic clusters-based classification and CINSARC for either OS or MFS (the clinical endpoint for which CINSARC demonstrated predictive capacity) estimation could not be carried out employing our study cohort samples.

We tested how CINSARC overlaps the lists of differentially expressed genes of each of the transcriptomic subtypes. We observed an overlap of 38% between the CINSARC gene set and the C1_under expressed genes and found a significant correlation between the enrichment scores of C1_under and CINSARC (Spearman’s Rank correlation = 0.78 between NES C1 under and CINSARC (Supplementary Figure S12)).

**Figure 4:**
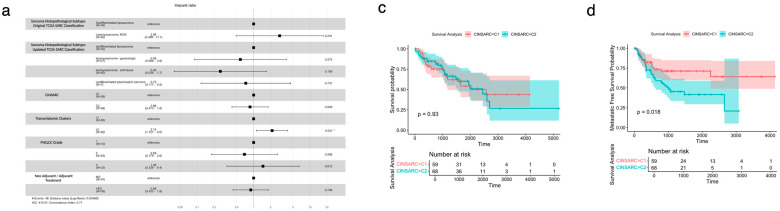

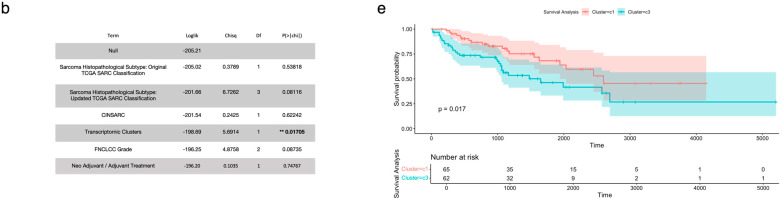
The transcriptomic cluster-based classification outperforms the CINSARC expression-based signature in terms of OS predictive capacity, as assessed by Cox Proportional Hazards Models, ANOVA tests, and Kaplan–Meier analyses with log-rank tests. (**a**) Forest plot showing the results of the evaluation of the impact of histopathological classification, CINSARC, transcriptomic clusters-based classification, and the FLNCC grade on OS considering the patients of the TCGA-SARC cohort after their classification according to CINSARC. (**b**) Table displaying the results of the ANOVA test applied to the Cox Proportional Hazards Model to assess the predictive ability of different variables for OS estimation using data from the TCGA-SARC (after the classification of TCGA-SARC patients according to CINSARC). (**c**) Survival analysis of the patients of the TCGA-SARC cohort after their classification according to CINSARC: OS analysis by the Kaplan–Meier method and respective curves (time scale is shown in days). (**d**) Survival analysis of the patients of the TCGA-SARC cohort after their classification according to CINSARC: MFS analysis by the Kaplan–Meier method and respective curves (time scale is shown in days). (**e**) Survival analysis of the patients of the TCGA-SARC cohort after their classification in accordance with the transcriptomic clusters-based classification: OS analysis by the Kaplan–Meier method and respective curves (time scale is shown in days). ** *p*-value <0.01; * *p*-value <0.05 and >0.01; # Number of.

Considering the TCGA-SARC patients (and after classifying them using CINSARC), an analysis using a Cox Proportional Hazards Model, including the histopathological classification (either the originally used in TCGA-SARC and the recently proposed and currently used one), CINSARC, transcriptomic clusters-based classification, FLNCC grade, and the use of neoadjuvant/adjuvant treatment was performed. The results revealed that the transcriptomic clusters-based classification was the only variable that showed a statistically significant correlation with OS, with C3 showing a negative prognostic effect (HR 2.13; 95% CI 1.121–4.0; *p* = 0.021) (Figure 4a). A subsequent ANOVA test of the Cox Proportional Hazards Model showed, once again, that the transcriptomic cluster-based classification was the most significant predictor of OS (*p* = 0.017) (Figure 4b).

Additionally, an OS analysis by the Kaplan–Meier log-rank test was performed for the CINSARC classified patients (C1 and C2) and showed no statistically significant differences (log rank *p* = 0.930) (Figure 4c). On the other hand, CINSARC has the ability, as previously reported, to distinguish between C1 and C2 in terms of metastasis free survival (MFS), displaying a log rank of *p* = 0.018 (Figure 4d).

In parallel, TCGA-SARC patients were also classified according to the transcriptomic clusters-based classification (as previously mentioned) and a survival analysis employing the Kaplan–Meier method showed, in this case, a significant difference in OS between C1 and C3 (log rank *p* = 0.017) (Figure 4e).

These findings confirm that, despite being able to accurately predict MFS, CINSARC does not have the capacity to differentiate distinct OS profiles within STS patients and displays a lower OS predictive capacity than the transcriptomic clusters-based classification.

### 3.9. DNA Alterations Analysis Reveals Unique Actionable Targets in the Transcriptomic Subtypes

**Figure 5:**
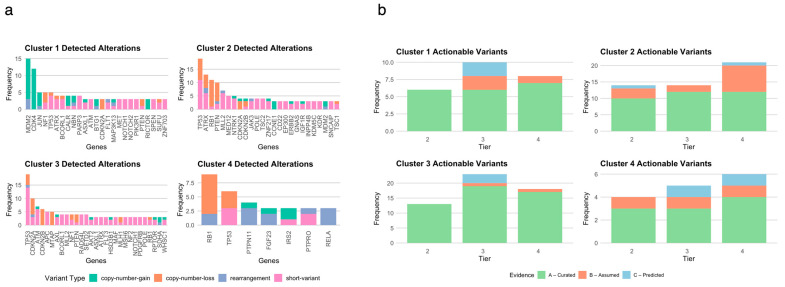
The analysis of the DNA alterations detected with FoundationOne^®^CDx for patients included in each of the four transcriptomic clusters/subtypes reveals unique actionable targets. (**a**) Frequency and types of genomic alterations detected by FoundationOne^®^CDx for patients included in each of the transcriptomic clusters/subtypes. (**b**) Distribution of the detected genomic alterations classified with MTBP per tier of actionability (Tier 2—Investigational, Tier 3—Hypothetical Target: Alteration-drug match is associated with antitumor activity, but the magnitude of benefit is unknown (potential cancer-repurposing opportunity, Tier 4—Hypothetical Target: pre-clinical evidence of actionability) and per functional relevance evidence for the alteration (A—Curated; B—Assumed; C—Predicted) for each of the transcriptomic clusters.

We analyzed the DNA alterations detected by FoundationOne^®^CDx in the patients included in each of the four transcriptomic clusters/subtypes (see Section 2). The frequency and types of the detected genomic alterations are represented in Figure 5a. An extensive description of these genomic alterations, and their respective distribution per transcriptomic subtype, is provided in Supplementary Material.

Then, we used MTBP [31] to systematize and interpret the functional and predictive value of each of the genomic variants that were found for the patients included in each of the transcriptomic subtypes (Tables S3–S6). The functional classification and the actionability tiering for gene variants performed by MTBP follow the ESMO Scale of Clinical Actionability for Molecular Targets (ESCATs). The distribution of the detected genomic alterations per tier of actionability and level of evidence for each of the transcriptomic clusters is shown in Figure 5b.

Overall, 151 gene variants classified with ESCAT evidence tiers ranging from 2 to 4 have been identified among the patients included in the study cohort (29 clinically actionable gene variants have been identified for patients included in C1, 51 for patients included in C2, 56 for patients included in C3, and 15 for patients included in C4). C2 displayed the highest number of gene variants classified with an ESCAT evidence tier 2 (15 variants), followed by C3 (14 variants). C3 showed the most significant number of gene variants classified either with an ESCAT evidence tier 3 (24 variants) or with an ESCAT evidence tier 4 (18 variants). C4 presented the lowest number of gene variants classified with an ESCAT evidence tier 2 (four variants), with an ESCAT evidence tier 3 (six variants) and with an ESCAT evidence tier 4 (five variants).

Tables S3–S6 display, extensively and in full detail, the particular features of each of the gene variants that were found and their distribution per transcriptomic cluster/subtype and per ESCAT evidence tier.

Among the complete pool of gene variants classified with an ESCAT evidence tier 2, there is a ubiquitous presence (across all the four clusters) of *MDM2* amplifications (conferring sensitivity to Brigimadlin and Milademetan) and a vast plethora of *TP53* alterations (mainly missense mutations conferring sensitivity to Pazopanib and Vorinostat). *MTAP* deletions (conferring sensitivity to MRTX1719 and AMG193) are also noteworthy, since they were found in three of the four clusters. *TSC2* mutations (conferring sensitivity to ABI-009) were also identified in two of the four clusters. Interestingly, *ERBB2* amplifications (conferring sensitivity to Trastuzumab Deruxtecan) (found in C2) and *PIK3CA* missense mutations (conferring sensitivity to Capivasertib and Copanlisib) (found in C3) were found in one of the four clusters.

C1 is marked, in terms of actionable alterations, by an enrichment in *MDM2* amplifications (Tier 2), *TP53* mutations (Tier 2), *NF1* mutations (Tier 3—conferring sensitivity to Selumetinib and resistance to Vemurafenib—and Tier 4—conferring sensitivity to Trametinib and Cobimetinib) *CDK4* amplifications (Tier 4), and alterations of different genes involved in HRR, namely, frameshift mutations of *RAD51B* (Tier 3), and missense mutations of *ATM* (Tier 3) and *BRIP1* (Tier 3), all of them conferring sensitivity to PARP inhibitors, namely, Olaparib.

C2 is characterized, besides *MDM2* amplifications (Tier 2) and *TP53* mutations (Tier 2), by *MTAP* deletions (Tier 2), *TSC2* mutations (Tier 2), *ERBB2* amplifications (Tier 2—conferring sensitivity to Trastuzumab Deruxtecan—and Tier 3—conferring sensitivity to a wide array of anti-HER2 agents, either in monotherapy or in combination with other drugs belonging either to the same anti-HER2 class or to other classes) and specific genetic alterations that are targets for tumor agnostic treatment approaches, such as *RET* missense mutations (Tier 3, which confer sensitivity to Selpercatinib and Pralsetinib). *POLE* missense mutations (Tier 3), that typically lead to a hypermutated and immunosensitive phenotype, conferring sensitivity to immune-checkpoint inhibitors such as Pembrolizumab, and *FGFR1* mutations (Tier 3—conferring sensitivity to Pemigatinib—and Tier 4—conferring sensitivity to Erdafitinib and AZD4547) are also of note.

C3 is portrayed by *MDM2* amplifications, *TP53* mutations, *MTAP* deletions, *TSC2* mutations, and *PIK3CA* mutations in terms of Tier 2 alterations. This subtype is particularly fertile in terms of actionable alterations. A mention should be made to *POLE* missense mutations (Tier 3, conferring sensitivity to Pembrolizumab), *KRAS* missense mutations (Tier 3 and Tier 4, conferring sensitivity and resistance to a plethora of different agents), *NRAS* missense mutations (Tier 3 and Tier 4, conferring sensitivity and resistance to a plethora of different agents), *MET* amplifications (Tier 3, conferring sensitivity to Capmatinib, Tepotinib, Telisotuzumab Vedotin and Crizotinib), *PTEN* frameshift mutations (Tier 3—conferring sensitivity to Capivasertib and Fulvestrant—and Tier 4—conferring sensitivity to Ipatasertib, GSK26364771, AZD8186), *VHL* missense mutations (Tier 3—conferring sensitivity to Everolimus), *CDK4* amplifications (Tier 4—conferring sensitivity to cyclin-dependent kinases inhibitors), *CDKN2A* mutations (Tier 4—conferring sensitivity to cyclin-dependent kinases inhibitors), and alterations of genes involved in the HRR mechanisms, namely, *ATM* (Tier 3) and *ATR* (Tier 3), conferring sensitivity to PARP inhibitors.

C4 is the subtype with the smallest number of actionable gene alterations among the four subtypes. *MDM2* amplifications (Tiers 2, 3 and 4), TP53 mutations (Tiers 2, 3 and 4), *MLH1* missense mutations (Tier 3, conferring sensitivity to PARP inhibitors), *BARD1* missense mutations (Tier 3, conferring sensitivity to PARP inhibitors) and *CDK4* amplifications (Tier 4, conferring sensitivity to cyclin-dependent kinases inhibitors) are found in this subtype.

Overall, there is a profusion of targetable alterations scattered across the different transcriptomic subtypes. Either alterations that are compelling targets for tumor-agnostic treatment approaches, such as the *RET* mutations and *ERBB2* amplifications found in C2, or alterations that are linked with defective DNA repair mechanisms, such as the mutations of distinct genes involved in HRR verified in C1, C3 and C4 and the mutations of *POLE* documented in C2 and C3, and a vast array of other specific alterations (some of them never previously documented in DDLPS, LMS and UPS) confer sensitivity to a broad spectrum of different agents.

### 3.10. RNA-seq Detected Fusions That Were Not Identified by DNA Sequencing (DNA-seq)

FoundationOne^®^RNA detected fusions that were not detected by FoundationOne^®^CDx in 9.1% of the cases (2/22) for which both DNA-seq and RNA-seq for rearrangement detection were clinically reportable. The inclusion of a high number of archival samples > 2 years old contributed to the high rate of absence of passage of the particularly rigorous post-sequencing QC metrics required for clinical RNA rearrangement detection. On the other hand, the great majority of samples (75/102; 73.5%) passed the QC metrics required for RNA-seq expression analysis (see Section 2). The disparity between these QC passage rates for the optimal detection of cancer-related gene fusions and rearrangements (318 gene panel) test and the gene expression profiling (1517 gene panel) test covered by FoundationOne^®^RNA lies in the different nature of each of these tests: the test designed for optimal detection of cancer-related gene fusions and rearrangements for 318 genes is a test developed for clinical use and, therefore, employs specially stringent QC criteria, while the gene expression profiling test for 1517 genes is a test designed for research use only and employs less strict QC criteria (see Section 2).

The STS histotypes that were provided for this study are not typically translocation-associated types; therefore, the limited number of detected fusions is relatively unsurprising.

An *HMGA2* (intron 3)::*TPH2* (intron 8) fusion was found in a case of DDLPS. This fusion was not detected in DNA because the *HMGA2* and *TPH2* genes are not baited on the FoundationOne^®^CDx gene panel. A *NOTCH3* (intron 24)::*BRD4* (intron 11) fusion was found in a case of UPS. Similarly, this fusion was not detected by FoundationOne^®^CDx because, while the exonic regions of both genes are covered on FoundationOne^®^CDx, the breakpoints for both genes occurred in intronic regions which are not covered.

Thus, RNA-seq provided additional value to DNA-seq by detecting reportable fusions.

## 4. Discussion

In this study, the analysis of RNA-seq data from a cohort composed of 102 samples of the three most common STS subtypes using unsupervised machine learning models allowed for the discovery of previously unknown molecular patterns and permitted the identification of four well-defined transcriptomic clusters, corresponding to four molecular/transcriptomic subtypes. This transcriptomic cluster/subtype-based classification has a clear prognostic value, which was externally validated. The prognostic value of this transcriptomic cluster/subtype-based classification is superior to currently used clinical-based prognostication tools (such as SARCULATOR nomograms) and to modern gold-standard molecular-based prognostication tools (such as CINSARC). The analysis of DNA-seq data from the same cohort of STS samples revealed unique and, in some cases, never documented molecular targets for precision treatment across different transcriptomic subtypes.

Clustering, the concept of grouping samples/patients based on the co-occurrence of molecular alterations, has been previously used to systematically analyze complex data generated by bone and soft tissue sarcoma molecular characterization approaches, allowing for the identification of specific sarcoma molecular clusters with particular clinical behaviors [33,36,37,38,39]. Consensus clustering has also already been used in two studies to identify STS molecular clusters [20,40] (Supplementary Table S7). Besides differences in terms of relative representation of distinct STS histotypes, molecular profiling approaches, methodological strategies for data analysis (combination with other types of unsupervised clustering or with different methods) and respective results, the main distinctive feature of our approach is its clinical-driven nature. Our method has been primarily developed using analytical tools developed for research use, but subsequently powered with analytical tools with a proven clinical utility. Moreover, it has included an extensive gathering of clinical variables, allowing for a better portrayal of the clinical significance of the transcriptomic subtypes and its defining molecular features. We used sequencing tests that have been developed for research and/or clinical use, are cost-effective and are, therefore, potentially useful in the clinical practice routine. These tests were used to analyze all the samples of the study cohort, solely for the purpose of this study, differing from an approach comprising the analysis of a previously constructed public database. Ultimately, we have identified gene expression signatures that display both a superlative prognostic and a potential predictive value, supreme indicators of clinical significance and impact. These gene expression signatures were identified following the use of consensus clustering to analyze data obtained from the application of a targeted-sequencing test whose gene set is composed by cancer-related genes, which may confer specificity to our findings (even considering that the gene set of this targeted sequencing test has not been primarily designed to cover a particular panel of genes whose differential expression profile is characteristic of STS).

The conceptual robustness of the identified transcriptomic clusters/subtypes is supported by the methodological approach (use of the Elbow method and verification of subtypes persistence with the removal of UPS samples from the samples pool), the presence of all of the included STS histopathological subtypes in each of the transcriptomic subtypes, and the distinct intrinsic nature of the molecular features that define each transcriptomic subtype.

Various of these cluster/subtype defining molecular traits are, themselves, novel and constitute, in some cases, breakthrough findings in STS (Supplementary Table S8 [20,41,42,43,44,45,46,47,48,49,50,51,52,53,54,55]).

Some of these particular molecular characteristics have never been previously reported in STS. The over expression of specific MAGE genes (*-A12*, *-A2B*, *-A3*, *-B1*, *-B2*, and -*C2*), other than *MAGE-A4* [43,44], verified in C2, and the over expression of HLA class II genes (*HLA-DMA*, *HLA-DMB*, *HLA-DOA*, *HLA-DQA*, *HLA-DRA* and *HLA-DRB1*), other than HLA class I genes [48,49], verified in C3 fall into this category.

Other subtype defining molecular alterations have already been reported in STS as exceedingly rare findings. The under expression of several genes involved in HRR mechanisms, potentially leading to homologous recombination deficiency (HRD), in C1 is an example [33,41]. The over expression of *SSX* genes (−1, −2, −2*B* and −3) documented in C2 is another illustrative case, especially considering that the sample pool did not include synovial sarcoma samples (even though the over expression of *SSX* genes may also be found in other STS histotypes, with a significant fraction of these STSs co-over expressing more than one *SSX* family member [44,45,46]).

From another angle, the coexistence of some of these subtype-specific molecular traits has not been previously described in STS. The coexistence of over expression of *CDK4* and under expression of genes involved in HRR, as verified in C1, is exemplifying. Even though the concomitant over expression of *MAGE* and *SSX* genes has already been reported in colorectal cancer [47], the simultaneous over expression of the prementioned specific MAGE genes and the aforesaid SSX genes, as verified in C2, has never been reported in the STS histotypes that compose our cohort. Supplementary Table S8 lists distinctive molecular traits per subtype, and a conceptual framing of its rarity or novelty based on a literature review.

Besides the originality of the molecular features that are the backbone of each subtype, the biological and clinical relevance of the transcriptomic subtypes also lie in their prognostic value. Three of the four identified subtypes within our cohort have a clear significant impact on OS, and the associated molecular signatures show, when compared with the histopathological classification and other variables, a better ability to predict OS, a finding that was externally validated with the TCGA-SARC cohort. Moreover, two of the four identified subtypes within our cohort have a significant impact on DFS and our transcriptomic cluster/subtype-based classification also shows, when compared with the histopathological classification and other variables, an exquisite ability to predict DFS. External validation with the TCGA-SARC cohort confirmed the transcriptomic cluster/subtype-based classification as the model with the best accuracy for DFS prediction. As a matter of fact, this transcriptomic cluster/subtype-based classification is an exquisite instrument to predict both OS and DFS allowing for accurate transversal and period-specific prognosis estimation in different settings of the natural history of oncological disease.

Besides its intrinsic prognostication power, our transcriptomic subtype-based classification, which was not originally designed or trained to specifically predict OS, exceeded the SARCULATOR nomograms OS predictive capacity and, when combined with age, displayed the strongest OS predictive ability among different individual variables (including SARCULATOR) and combinations of variables in an analysis within the study cohort. This was independently validated using the TCGA-SARC cohort, even considering the marginal difference verified between the prognostic accuracy of the transcriptomic subtype-based classification and SARCULATOR. Additionally, our transcriptomic subtype-based classification displayed, in an analysis using the TCGA-SARC cohort, a superior DFS predictive capability than the other molecular-based prognostic signature, namely, CINSARC (which is a best in test predictor of MFS). This finding raises the possibility that the transcriptomic cluster/subtype-based classification we have identified may have a better capability to predict not only death, but also local recurrence than CINSARC.

Moreover, the present study cohort includes, up to a certain point, STS populations that were underrepresented in the cohorts used for the development of the past mentioned nomograms, including eSTS, RPS and trunk STS concomitantly, a small number of patients treated in a neoadjuvant context (3/101) and a small number of patients with an unresectable RPS (2/101). Furthermore, our results show that the use of molecular data, that may be obtainable from the sequencing of a biopsy specimen, or the combination of a variable that is objective and independent from a histopathological examination, age, and molecular data, is superlative, making this prognosis-estimation strategy potentially employable in a preoperative setting, in contrast to the available nomograms as some of the variables included for their calculation are not available before surgery [56]. Finally, our molecular-based classification may refine SARCULATOR’s prognosis assessment, allowing for prognostic sub-stratification within specific SARCULATOR-defined prognostic groups in the study cohort. This was not verified when the TCGA-SARC cohort was used for validation potentially because of the different preponderance, in comparison with the study cohort, of patients with a grade 3 STS that display a predicted 5-year OS ≤ 60% in this cohort, as reported in Section 3.

Our transcriptomic clusters/subtype-based classification is, as CINSARC, an expression-based signature established from the analysis of primary non-translocation-related STS [24,35]. A comparative analysis of the prognostic value of these two STS molecular-based signatures whose prognostic value outperformed the histopathological-based grading system in STS is essential. The overlap between the CINSARC gene set and the C1 under expressed genes and the significant correlation between the enrichment scores of C1_under and CINSARC is not surprising, considering the molecular features that characterize CINSARC expression (67 genes involved in the control of chromosome integrity and mitosis; CINSARC’s expression is associated with genomic and chromosomal instability [24,35]) and the molecular features that characterize C1_under (under expression of genes involved in HRR, potentially leading to HRD and chromosomal instability).

Methodologically, there are critical differences between the approach we employed for the development of our transcriptomic cluster/subtype-based classification and the approach used for the development of CINSARC, as explained in detail in Supplementary Material.

Apart from the methodological dimension, the nature of the clinical endpoints that may accurately be estimated following the use of CINSARC or our transcriptomic cluster/subtype-based classification is also distinct. The OS estimation capacity of our transcriptomic cluster/subtype-based classification clearly surpasses CINSARC in a head-to-head comparison using the TCGA-SARC cohort, as shown in Section 3. Additionally, the DFS estimation capacity of our transcriptomic cluster/subtype-based classification is superior to the CINSARC DFS estimation accuracy in another analysis using the TCGA-SARC cohort, as previously mentioned and as shown in Section 3. Our analysis also shows that, using the same cohort, while CINSARC accurately differentiates patients with different MFS profiles, it does not have the power to discriminate groups of patients with different OS profiles, which is something that our transcriptomic cluster/subtype-based approach is capable of.

Past series, either in a real-world conventional practice context [18] or in an investigational context [57] have demonstrated that a significant percentage of STS display druggable molecular alterations and that both STS patients treated using a molecular-guided personalized treatment in a conventional context [18] and STS patients enrolled in biomarker-matched early-phase clinical trials [57] show a significant benefit from the employment of a molecular-guided strategy.

The analysis of DNA-seq data of patients included in each transcriptomic subtype highlighted 151 actionable gene variants, comprising alterations that are putative targets for tumor-agnostic treatments and alterations that are targets for tumor-specific approaches, conferring sensitivity to a variety of molecularly targeted agents (MTAs) and new antineoplastic drug classes. While the most broadly represented genes for which alterations were found across different transcriptomic subtypes overlap genes for which alterations have more commonly been reported in another series (i.e., *TP53*, *MDM2* and *PIK3CA*) [18], we managed to identify alterations and targets whose existence in STS has been under reported or never documented (i.e., *ERBB2* amplification, *MET* amplification, *POLE* mutations, *RET* mutations, *KRAS* and *NRAS* mutations). Notably, RNA-seq identified two fusions not detected using DNA-seq (*HMGA2*::*TPH2* in a case of DDLPS and *NOTCH3*::*BRD4* in a case of UPS) despite RNA-seq QC metrics being sufficient for rearrangement detection in only a small percentage of samples (*n* = 22).

Besides the actionable gene variants that have been identified following the analysis of the DNA-seq data of patients included in each transcriptomic subtype, the specific gene and pathway expression patterns that uniquely define each subtype may also provide clues about putative translational approaches to validate presumable particular treatment sensitivities of each transcriptomic subtype. Ultimately, this could shed light on the way treatment could be guided according to the proximity of STS transcriptomic profiles to the transcriptomic signatures that define each transcriptomic subtype. C1 (DNA repair-deficient/HRD-like/Hypermutant) most likely displays sensitivity to PARP inhibitors and DNA damage-targeting agents (as well as to cyclin-dependent kinases (CDKs) inhibitors, such as CDK 4/6 inhibitors, and to MDM2 antagonists), and, therefore, those should be the type of agents that should be preferentially evaluated in the context of a clinical trial for patients whose STS transcriptomic profile is close to C1. Similarly, C2 (Cancer testis antigens-enriched/Immunogenic) potentially shows a particular sensitivity to cancer testis antigens-directed immunotherapies, namely, cancer testis antigen-directed T cell receptor therapies and cancer testis antigen-directed vaccines. C3 (HLA-high/Immune Activated) may display an increased sensitivity to different types of immune-checkpoint inhibitors and other types of immunomodulators. C4 (Claudin-high/Structural) may be amenable to be treated with structural/adhesion-targeting strategies, such as claudin-directed agents in development. A prospective, multicenter clinical trial will be needed to validate both the prognostic (guiding the decision whether to perform adjuvant treatment or not) and the predictive (guiding the decision of which drug should be used to treat advanced disease) utility of this classification and of these molecular signatures in STS. Our group is actively structuring the development of a clinical trial with those purposes.

While the great majority of patients included in past STS molecular-profiling series were patients in an advanced setting (most of them heavily pretreated in a metastatic context), the vast bulk of the patients (95%) included in our series presented with localized disease. Nevertheless, real-world series also included patients without metastases at the time of their case discussion in an MTB (15%) [18], having recommended the addition of MTA to a conventional chemotherapy backbone in some of the patients with actionable alterations and an early-stage disease setting (2 out of 10 patients) [18] and used, as we did in our series, primary tumor samples for molecular profiling (50% of included patients) [18] with similar conceptual results. A recent groundbreaking study characterized the genomic differences between early-stage untreated primary tumors and late-stage treated metastatic tumors [58]. This study included primary and metastatic samples of LMS (15 vs. 47) and liposarcoma (17 vs. 25) [58]. No significant variations in clonality, karyotype, mutational burden, mutational signature profile, total number of driver gene alterations, frequency of therapeutically actionable gene variants and treatment-associated driver genes were found between primary and metastatic samples of both LMS and liposarcoma, but one cannot ignore that exposure to treatment (either chemotherapy or radiotherapy) potentially further scars the tumor genome and introduces an evolutionary bottleneck that may select for therapy-resistant drivers [58], making molecular profiling of metastatic lesions in an advanced setting recommendable.

This study has some important limitations that are important to underline. The retrospective nature of the study should be taken into account. This study cohort is single-centered (even though it includes patients with different ethnical ancestries and backgrounds—European and African-native patients). In addition, this study population and samples pool are composed of a limited number of three STS histopathological subtypes (not comprising, for instance, ultra-rare subtypes, for which the relevance and usefulness of molecular signatures with prognostic and predictive value such as the ones that we have identified could be even greater; however, this methodological approach can and should be replicated in a cohort composed of ultra-rare STS samples), and is composed of primary tumor samples of a great majority (95%) of STS patients with early stage/localized disease. Therefore, there is not a significant representation either of samples from STS metastases or of patients with advanced STS. Moreover, the samples included in each of the batches that were sent to Foundation Medicine for DNA-seq and RNA-seq were collected in different timepoints and, therefore, display heterogenous chronological ages (a fact that directly impacts the differential likeability of degradation of the biological material and the distinct quality of the samples for the planned sequencing analysis). In the same line, the degree of degradation of the samples that were analyzed and, subsequently, the amount of samples for which the quality control for DNA-seq and RNA-seq was not successful, namely, in the context of fusion/splice site detection with RNA, is also a limitation.

On the other hand, the sequencing tests that were used for molecular profiling are targeted sequencing tests, which offer results with a distinct conceptual coverage than the ones that could be offered by a whole genome or whole exome sequencing approach.

Although the gene set of this targeted sequencing test (namely, Foundation One^®^ RNA) has not been primarily created specifically using a particular panel of genes whose differential expression profile portrays and is characteristic of STS, it covers 52% of the genes that comprise the gene set of the single molecular signature that is based on specific gene expression profiling in STS (CINSARC), and shows an overlap of 38% between the pattern of expression of a specific array of covered genes and the pattern of expression of the genes that compose CINSARC’s gene set (with a Spearman’s rank correlation of 0.78). The analysis of the gene expression data obtained following the use of this targeted sequencing test to study STS samples, allowed for the identification of a new classifying tool (based on molecular subtypes with distinct biological and behavioral profiles that emerged from the analysis of gene expression profiling) that displays a provenly superior OS and DFS predictive capacity and prognostic value when compared with CINSARC (a finding that has been externally validated using the TCGA-SARC dataset), being one of the first molecular-based classifications with OS predictive capability in STS.

## 5. Conclusions

We performed DNA-seq and RNA-seq to profile a cohort composed of more than 100 high-grade STS samples of three of the most common STS histotypes. RNA-seq data was analyzed using unsupervised machine learning models, uncovering previously unknown molecular patterns and unravelling four distinct transcriptomic subtypes with clear prognostic value (displaying notable OS and DFS estimating capacity). The transcriptomic cluster/subtype-based classification outperformed both currently employed clinical-based tools (SARCULATOR nomograms) and gold-standard molecular-based methods (CINSARC) in prognostic accuracy (superior OS predictive capability than SARCULATOR and CINSARC, and superior DFS predictive capability than CINSARC), being one of the first molecular-based classifications capable of predicting OS in STS. Moreover, DNA-seq data was scrutinized revealing unique and previously unreported molecular targets across transcriptomic subtypes, highlighting opportunities for precision treatment.

This new classification tool has the potential to provide superior prognostic value and to be able to identify novel molecular targets for precision treatment, possibly representing a cutting-edge tool for predicting prognosis and guiding treatment across different stages of STS.

Therapeutic intervention, guided and framed by the described transcriptomic cluster-based prognosis profiling and molecular targets identification, should be prospectively tested in a multicentric clinical trial.

## Data Availability

Different data and generated datasets have been deposited in figshare under the following URL: https://figshare.com/s/6a70cbb12d2738a6e60b (accessed on 15 October 2024) (to be made public upon publication). Code Availability: All code is available at https://github.com/QuantitativeBiology/Sarcoma-TC-Clusters (accessed on 15 October 2024).

## Supplementary Materials

The following supporting information can be downloaded at: https://www.mdpi.com/article/10.3390/cancers17172861/s1. Figure S1: Consensus clustering analysis: Optimal number of clusters. Consensus matrix (1a) and Delta area plot (1b); Figure S2: Comparison of differentially expressed genes between pairs of clusters (Volcano plots); Figure S3: Transcriptomic clusters/subtypes and their respective molecular signatures exquisite prognostic value, assessed by a Cox Proportional Hazards Models including age and treatment modality as variables; Figure S4: ssGSEA Normalized Enrichment Score for each TCGA-SARC patient (Heatmap Plot). The heatmap plot show a significant enrichment of these patients samples to c1_under and c3_over; Figure S5: Transcriptomic clusters/subtypes and their respective molecular signatures exquisite prognostic value in terms of DFS. (a) Forest plot showing the results of the evaluation of the differential impact of distinct demographical, clinical, histopathological and molecular variables on DFS in the study cohort using a Cox Proportional Hazards Model. (b) Forest plot showing the results of the evaluation of the differential impact of different variables (including age and treatment modality) on DFS in the study cohort using a Cox Proportional Hazards Model. (c) Table displaying the results of the ANOVA test applied to the Cox Proportional Hazards Model to assess the pre-dictive ability of different variables for DFS estimation in the study cohort. (d) Forest plot showing the results of the evaluation of the differential impact of distinct histopathological and molecular var-iables on DFS considering the TCGA-SARC patients (classified in accordance with the tran-scriptomic clusters-based classification) using a Cox Proportional Hazards Model. (e) Table dis-playing the results of the ANOVA test applied to the Cox Proportional Hazards Model to assess the predictive ability of different variables for DFS estimation in the validation cohort (TCGA-SARC); Figure S6: Survival analysis (using the Kaplan-Meier method) of the TCGA-SARC (external cohort) patients classified per transcriptomic cluster/subtype (C1 and C3): DFS analysis by the Kaplan-Meier method and respective curves; Figure S7: Persistence of ssGSEA enrichment in C1_under and C3_over of the TCGA-SARC pop-ulation persist following the exclusion of UPS patients; Figure S8: Differential impact of distinct histopathological and molecular factors on overall survival considering TCGA-SARC patients, after the exclusion of UPS patients (Forest plot); Figure S9: Transcriptomic cluster-based classification, and other clinical and molecular-based models’ prognostic performance in terms of DFS. Bar chart showing the concordance indexes of different prognostic models employed using the population of the validation cohort (TCGA-SARC) (including CINSARC, CINSARC + Age, SARC, TC, TC + Age, SARC + CINSARC, SARC + TC, SARC + TC + CINSARC); Figure S10: Survival analysis (using the Kaplan-Meier method) of the study cohort patients that have displayed either a 5-year OS ≤ 60% and a 5-year OS > 60% (Sarculator-based): OS analysis by the Kaplan-Meier method and respective curves; Figure S11: Survival analysis (using the Kaplan-Meier method) of the TCGA-SARC (external cohort) patients that have displayed either a 5-year OS ≤ 60% and a 5-year OS > 60% (Sarculator-based): OS analysis by the Kaplan-Meier method and respective curves. The generic analysis including DDLPS, LMS and UPS patients from the TCGA-SARC cohort is displayed on Supplementary Figure S11A: The specific analysis including DDLPS, LMS and UPS patients from the TCGA-SARC cohort and that display an STS with an FNLCC grade 3 are shown on Supplementary Figure S11B; Figure S12: Correlation between ssGSEA enrichment scores (NES) from c1_under, c3_over and CINSARC genes (Spearman correlation plot); Table S1: Demographic characteristics of the study population and main features of the included STS cases; Table S2: Surgical and systemic treatment details of the patients who were considered for a curative surgical approach at IPOLFG; Table S3: Actionable gene variants (distributed per ESCAT evidence tier) found for patients included in C1. For each variant, gene identification, nature of the alteration, functional relevance evidence for the alteration (A—Curated; B—Assumed; C—Predicted) and the predictive value of the alteration is provided (2—Investigational; 3—Hypothetical target: Alteration-drug match is associated with antitumor activity, but magnitude of benefit is unknown (potential cancer-repurposing opportunity); 4—Hypothetical target: pre-clinical evidence of actionability); Table S4: Actionable gene variants (distributed per ESCAT evidence tier) found for patients included in C2. For each variant, gene identification, nature of the alteration, functional relevance evidence for the alteration (—Curated; B—Assumed; C—Predicted) and the predictive value of the alteration is provided (2—Investigational; 3—Hypothetical target: Alteration-drug match is associated with antitumor activity, but magnitude of benefit is unknown (potential cancer-repurposing opportunity); 4—Hypothetical target: pre-clinical evidence of actionability); Table S5: Actionable gene variants (distributed per ESCAT evidence tier) found for patients included in C3. For each variant, gene identification, nature of the alteration, functional relevance evidence for the alteration (A—Curated; B—Assumed; C—Predicted) and the predictive value of the alteration is provided (2—Investigational; 3—Hypothetical target: Alteration-drug match is associated with antitumor activity, but magnitude of benefit is unknown (potential cancer-repurposing opportunity); 4—Hypothetical target: pre-clinical evidence of actionability); Table S6: Actionable gene variants (distributed per ESCAT evidence tier) found for patients included in C4. For each variant, gene identification, nature of the alteration, functional relevance evidence for the alteration (A—Curated; B—Assumed; C—Predicted) and the predictive value of the alteration is provided (2—Investigational; 3—Hypothetical target: Alteration-drug match is associated with antitumor activity, but magnitude of benefit is unknown (potential cancer-repurposing opportunity); 4—Hypothetical target: pre-clinical evidence of actionability); Table S7: Characteristics of the studies that also employed unsupervised consensus clustering to analyze data originated from STS molecular profiling approaches. This table provides, for each study, the STS histopathological subtypes of the samples that have been included, the types of molecular analyses that were performed (single or multi-omics, types of sequencing approaches that were used), the aims, methodological similarities and differences relative to our approach and, conceptually, the most relevant results; Table S8: Most relevant distinctive molecular features of each transcriptomic cluster and conceptual rarity or novelty/originality of each feature.

## Appendix A

**Table A1:** Distribution of differentially expressed genes (over and under expressed) per transcriptomic cluster.

	Genes	
	Over Expressed	Under Expressed
Cluster 1 DNA repair-deficient HRD-like/Hypermutant	*BCAM*, *CBFA2T3*, *CCL19, CCND2*, *CD79B, CDK4*, *CX3CL1*, *DTX1*, *ECSCR*, *ERG, FMOD*, *GRM4*, *NKD1*, *OLFM1*, *PAX5, PLVAP*, *PNOC*, *SOX18*, *TTYH1*, *ZBTB46*	*ABL2*, *ANLN*, *AURKA*, *AURKB*, *BRCA1*, *BRCA2*, *BRIP1*, *BUB1*, *BUB1B*, *CCNA2*, *CCNB1*, *CCNB2*, *CDC20*, *CDC25C*, *CDCA5*, *CDCA8*, *CDKN3*, *CENPF*, *CENPM*, *CEP55*, *CHEK1*, *CRNDE*, *DEK*, *ECT2*, *EPS15*, *EXO1*, *FANCD2*, *FGFR1OP*, *GINS2*, *GMNN*, *HIST1H3B*, *KIF23*, *KIF2C*, *MALT1*, *MCM4*, *MELK*, *NDC80*, *NEK2*, *NUF2*, *PALB2*, *PBK*, *PTTG1*, *RAC1*, *RAD51*, *RAD51AP1*, *RAD54L*, *RRAGC*, *RRM2*, *SNW1*, *STIL*, *TOP2A*, *TPX2*, *TTK*, *TYMS*, *UBE2C*, *UBE2T*, *WHSC1*, *XPO*
Cluster 2 Cancer testis antigens-enriched Immunogenic	*ACVR1C*, *BAP1*, *CTNNB1*, *FZD6*, *JAZF1, MAGEA12*, *MAGEA2B*, *MAGEA3, MAGEB1*, *MAGEB2*, *MAGEC2*, *MMP11, MRAS*, *RGS16*, *SSX1*, *SSX2*, *SSX2B*, *SSX3*	*DHX58*, *IL12A*, *LILRB5*, *MAP3K8*, *SULT1A1*, *TNFRSF1B*
Cluster 3 HLA-high Immune Activated	*ATIC*, *BATF*, *BTK*, *CCL18*, *CCL2*, *CCR5*, *CD3G*, *CD74*, *CD84*, *CSF2*, *CXCL10*, *CYBB*, *CYLD*, *ETV5*, *FAM26F*, *FCGR3B*, *FGR*, *FN1, FPR3*, *GBP5*, *GMFG*, *HAVCR2*, *HLA.DMA*, *HLA.DMB*, *HLA.DOA*, *HLA.DQA1*, *HLA.DRA*, *HLA.DRB1*, *HMGA1*, *IL21R*, *IL7R*, *ITGB2*, *JAML*, *KCNMA1*, *LAIR1*, *LCP1*, *NFKB2*, *PHF11*, *PLEK2*, *PSMB10*, *RGS10*, *SEMA7A*, *SERPINE1*, *SYK*, *TGFB1*	*AXIN2*, *BCL9*, *BMP4*, *CDKN1C*, *CDKN2A*, *CITED4*, *DCLK1*, *DHH*, *DOT1L*, *FGFR2*, *FGFR3*, *FOXC1*, *FOXO1*, *FOXO4*, *FOXO6*, *FZD7*, *GAS1*, *GPC4*, *HAP1*, *HES1*, *KDM5C*, *LINC00598*, *NRTN*, *PBX1*, *PDGFD*, *PHLPP1*, *PRKACG*, *SCUBE2*, *SEMA6D*, *SESN3*, *SH3PXD2A*, *SMAD9*, *TCF7L1*, *TCF7L2*, *TET1*, *TMEM38A*, *TP53INP2*, *TRIM2*, *WNT11*, *ZNF521*
Cluster 4 Claudin-high Structural	*ADRB2*, *CD34*, *CFD*, *CLCA2*, *CLDN4*, *DDR2*, *FAM64A*, *GAS7*, *IL6ST*, *LINC.ROR*, *PDGFD*, *PGR*, *PHLPP1*, *SMAD3*, *SYCP3, TEK*, *TP73*	*ACTN1*, *FZD2*, *HOPX*, *NBEAP1*
