# Supplementary material for: Transcriptomic-Based Classification Identifies Prognostic Subtypes and Therapeutic Strategies in Soft Tissue Sarcomas
Source: Cancers (Basel). 2025 Aug 30;17(17):2861. doi: 10.3390/cancers17172861 (PMC12427208; doi:10.3390/cancers17172861)
Supplement: Supplementary file 1 [file cancers-17-02861-s001.zip › cancers-3740065-supplementary.pdf]

cancers

MDPI

Supplementary Material

Results

Genes included in CINSARC 67-gene prognostic signature

ANLN, *ASPM, AURKA, AURKB, BIRC5, *BORA, BUB1, BUB1B, CCNA2, CCNB1, CCNB2, CDC20, CDC45, CDC6, *CDC7, *CDCA2, CDCA3, CDCA8, *CDK1, CENPA, *CENPE, *CENPL, CEP55, CHEK1, *CKAP5, *CKS2, ECT2, *ESPL1, *FANCI, *FBXO5, FOXM1, H2AFX, *HP1BP3, *KIF11, *KIF14, *KIF15, *KIF18A, *KIF20A, KIF23, KIF2C, *KIF4A, *MAD2L1, MCM2, *MCM7, MELK, *NCAPH, NDE1, NEK2, NUF2, *OIP5, PBK, *PLK4, *PRC1, PTTG1, RAD51AP1, *RNASEH2A, RRM2, *SGO2, *SMC2, *SPAG5, *SPC25, TOP2A, TPX2, TRIP13, TTK, UBE2C, ZWINT

Note: Genes included in the CINSARC 67-gene panel whose expression is not evaluated by F1RNA are marked with an asterisk (*).

Characterization of frequency and types of genomic alterations detected by FoundationOne®CDx in patients included in each transcriptomic cluster

Among the patients included in C1, the most frequently found gene alterations were amplifications, especially in the MDM2 gene, whose amplification was found in 15 cases. Amplifications were also frequently found in other genes, such as CDK4 (7 cases) and JUN (6 cases). On the other hand, copy-number-losses in NF1 were found in 4 cases, while short variants in TP53 were identified in 5 cases. The genomic profile of the patients included in C1 is mostly portrayed by a mixture of frequent copy-number-gains in MDM2, CDK4, and JUN, along with copy-number-losses in NF1 and short variants (point mutations) in TP53.

Among the patients that comprise C2, the most commonly identified gene alterations were short variants. TP53 was the gene where alterations were more frequently found (18 cases). ATRX and RB1 alterations were also frequently verified. RB1 copy-number-losses were observed in 10 cases. In parallel, PTEN copy-number losses were verified in 5 cases, and MED12 and NTRK1 alterations were observed in 4 and 3 cases, respectively. The genomic profile of the patients included in C2 is defined by frequent TP53 alterations of different types (mostly short-variants and copy-number-losses) and RB1 copy-number-losses, with an additional plethora of alterations in ATRX, PTEN, MED12, and NTRK1.

Amidst patients that integrate C3, the pattern of identified genomic alterations was largely marked by deletions (short variants and copy-number-losses), with TP53 displaying a high frequency of short variants (16 cases) (alongside APC (5 cases), MLH1 (4 cases), and RAD54L (3 cases)), while CDKN2A, CDKN2B and MTAP were frequently affected by copy-number losses. Concomitantly, NF1 deletions were observed in 6 cases. The genomic profile of the patients included in C3 is principally portrayed by a high frequency of deletions in CDKN2A/B, MTAP and NF1, alongside frequent point mutations in TP53 and other tumor suppressor genes.

Regarding patients that are part and compose C4, short variants have been often verified, more particularly in TP53 (7 cases) and PTPN11 (3 cases). Copy-number-losses have also been verified in RB1 (6 cases). Finally, FGF23 rearrangements are also common, being observed in 4 cases. The genomic profile of patients included in C4 is characterized by a combination of RB1 copy-number-losses, TP53 mutations (short variants and copy-number-losses), and frequent FGF23 and PTPN11 structural rearrangements.

Discussion

Differences between the methodological approach used for the development of the presented transcriptomic cluster-based classification and the methodological approach employed for the development of CINSARC

Methodologically, we employ ssGSEA to derive pathway enrichment scores from normalized gene expression data, encompassing both KEGG pathways and our custom gene sets. Following this, we extract p-values for each pathway and apply an adjustment as mentioned in **Methods**. This adjustment is performed both across patients and pathways, ensuring that the comparisons are robust and reducing the likelihood of false positives. After excluding KEGG pathways, samples are assigned to the pathway with the minimal adjusted p-value. In contrast, CINSARC classifies samples by calculating the distance between a sample's normalized gene expression data and the centroids of CINSARC gene set. Samples are then classified to the closest centroid. While the CINSARC approach relies on the proximity of a sample's gene expression to the nearest centroid within predefined gene sets (CINSARC C1 and C2), our method is based on adjusted ssGSEA enrichment scores. By incorporating KEGG pathways and adjusting p-values both across patients and pathways, our method provides a robust strategy of representing the enrichment, which is adjusted for pathway and sample-wise false positives.

Supplementary Figures

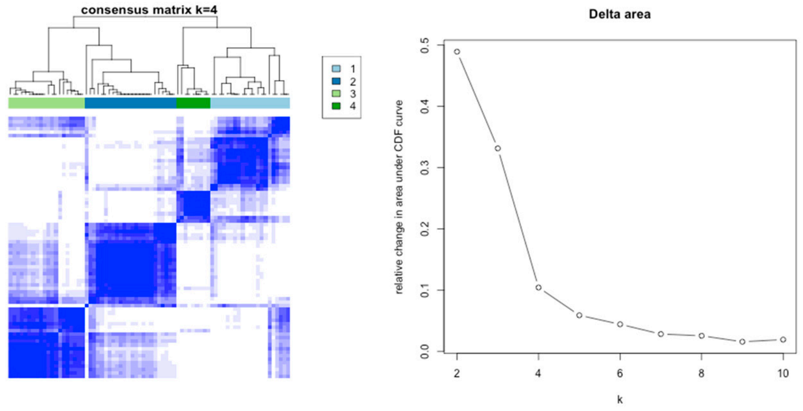

1a **1b**

Figure S1. – Consensus clustering analysis: Optimal number of clusters. Consensus matrix (1a) and Delta area plot (1b).

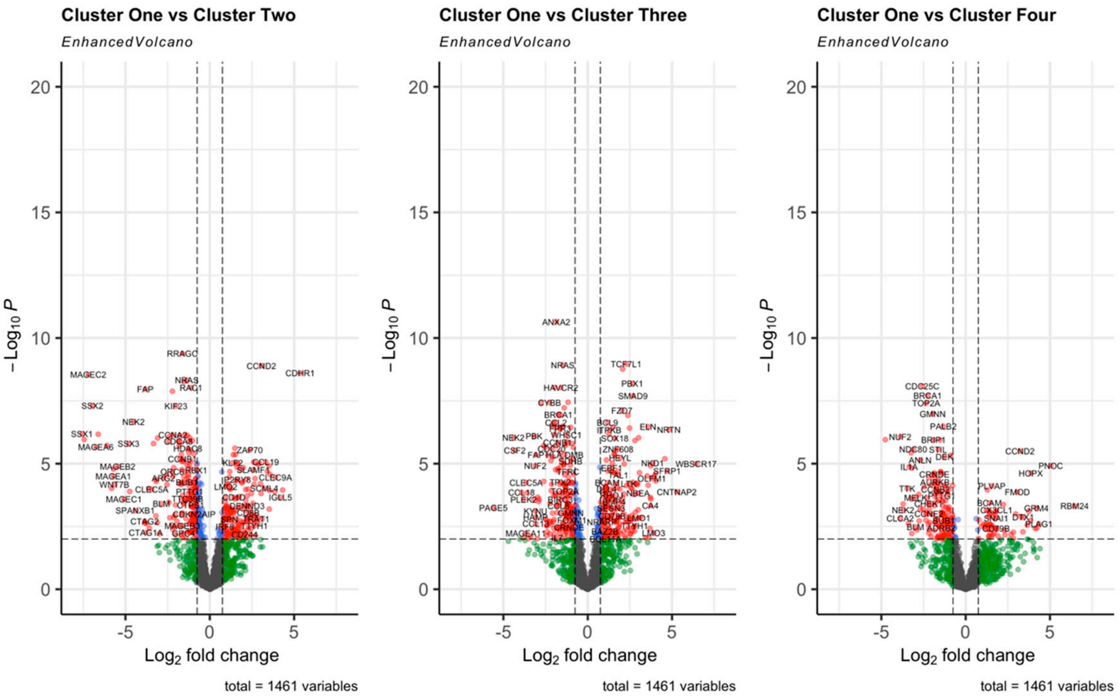

Figure S2. - Comparison of differentially expressed genes between pairs of clusters (Volcano plots).

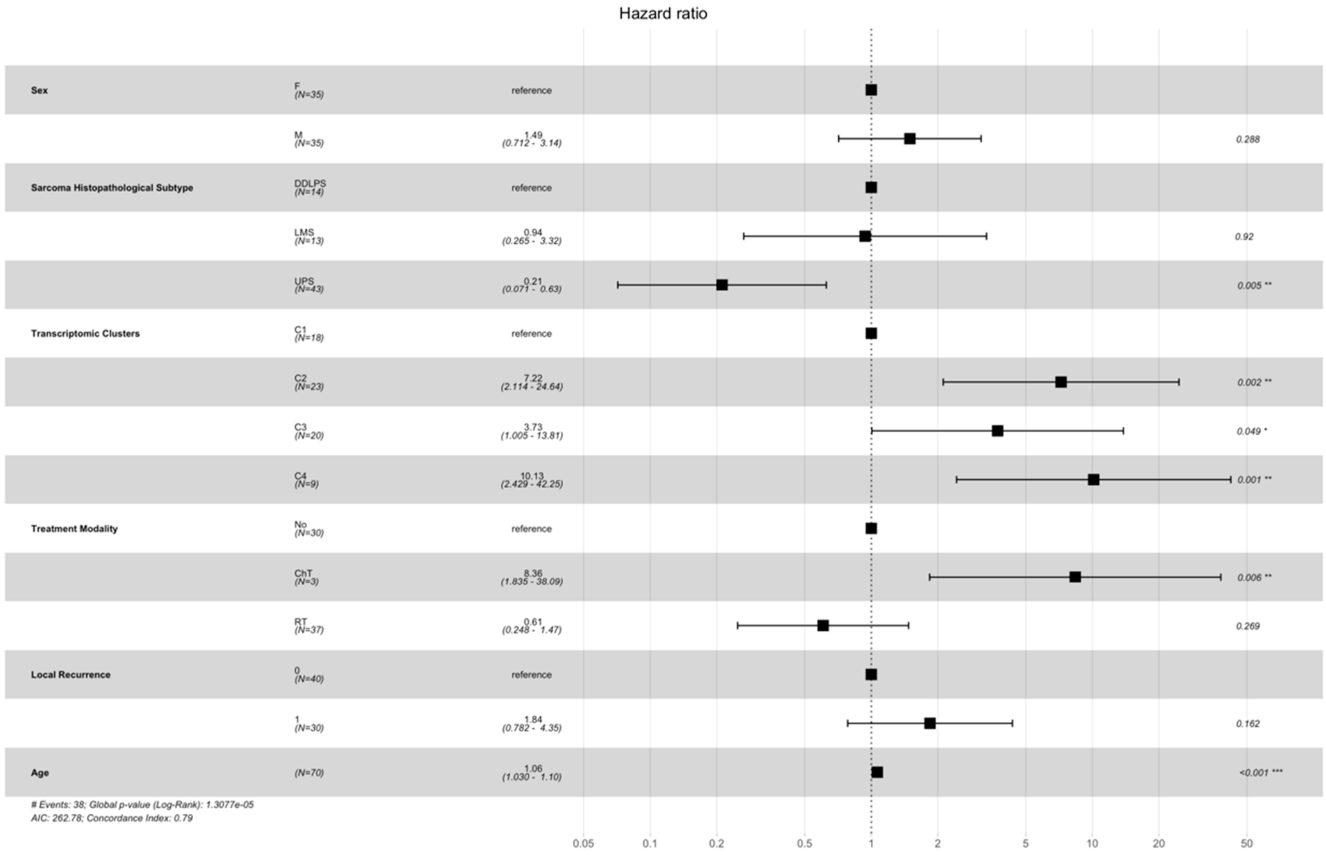

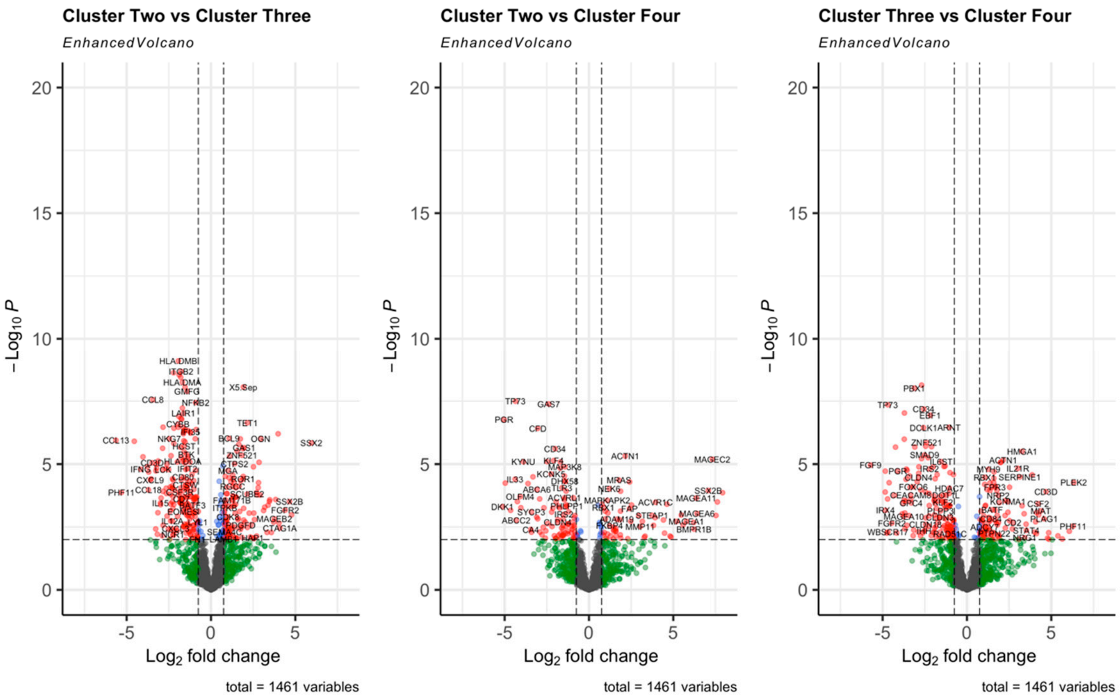

Figure S3. Transcriptomic clusters/subtypes and their respective molecular signatures exquisite prognostic value, assessed by a Cox Proportional Hazards Models including age and treatment modality as variables.

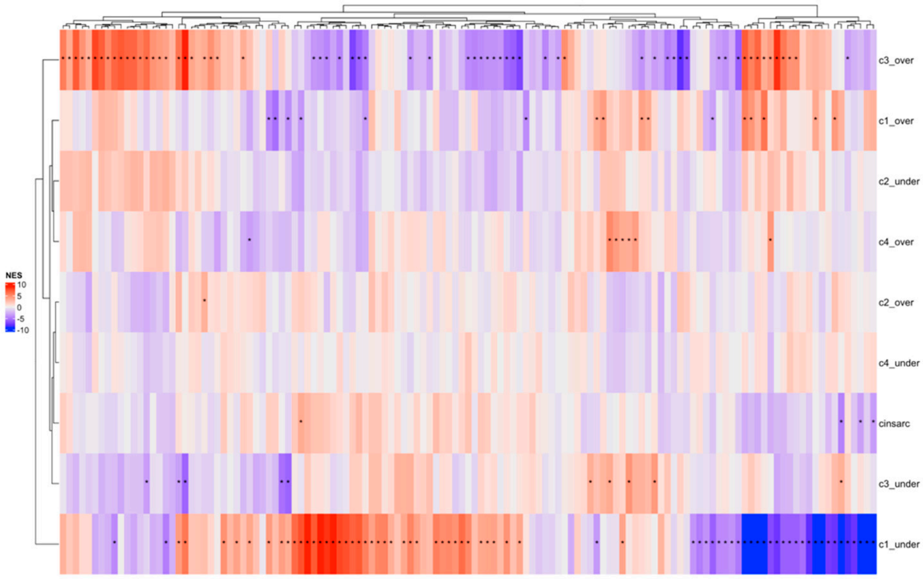

Figure S4. ssGSEA Normalized Enrichment Score for each TCGA-SARC patient (Heatmap Plot). The heatmap plot show a significant enrichment of these patients samples to c1_under and c3_over.

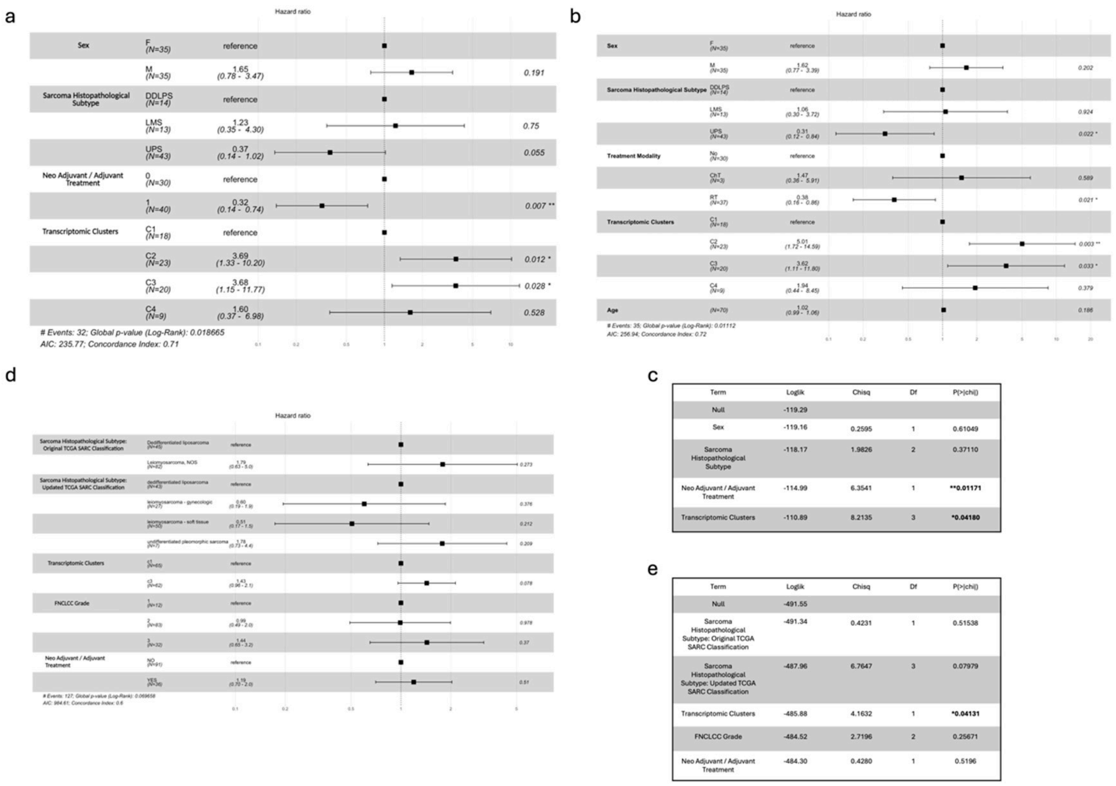

c

Term	Loglik	Chisq	Df	P(>|chi|)
Null	-119.29			
Sex	-119.16	0.2595	1	0.61049
Sarcoma Histopathological Subtype	-118.17	1.9826	2	0.37110
Neo Adjuvant / Adjuvant Treatment	-114.99	6.3541	1	****0.01171**
Transcriptomic Clusters	-110.89	8.2135	3	***0.04180**

e

Term	Loglik	Chisq	Df	P(>|chi|)
Null	-491.55			
Sarcoma Histopathological Subtype: Original TCGA SARC Classification	-491.34	0.4231	1	0.51538
Sarcoma Histopathological Subtype: Updated TCGA SARC Classification	-487.96	6.7647	3	0.07979
Transcriptomic Clusters	-485.88	4.1632	1	***0.04131**
FNCLCC Grade	-484.52	2.7196	2	0.25671
Neo Adjuvant / Adjuvant Treatment	-484.30	0.4280	1	0.5196

Figure S5. Transcriptomic clusters/subtypes and their respective molecular signatures exquisite prognostic value in terms of DFS. **a)** Forest plot showing the results of the evaluation of the differential impact of distinct demographical, clinical, histopathological and molecular variables on DFS in the study cohort using a Cox Proportional Hazards Model. **b)** Forest plot showing the results of the evaluation of the differential impact of different variables (including age and treatment modality) on DFS in the study cohort using a Cox Proportional Hazards Model. **c)** Table displaying the results of the ANOVA test applied to the Cox Proportional Hazards Model to assess the predictive ability of different variables for DFS estimation in the study cohort. **d)** Forest plot showing the results of the evaluation of the differential impact of distinct histopathological and molecular variables on DFS considering the TCGA-SARC patients (classified in accordance with the transcriptomic clusters-based classification) using a Cox Proportional Hazards Model. **e)** Table displaying the results of the ANOVA test applied to the Cox Proportional Hazards Model to assess the predictive ability of different variables for DFS estimation in the validation cohort (TCGA-SARC).

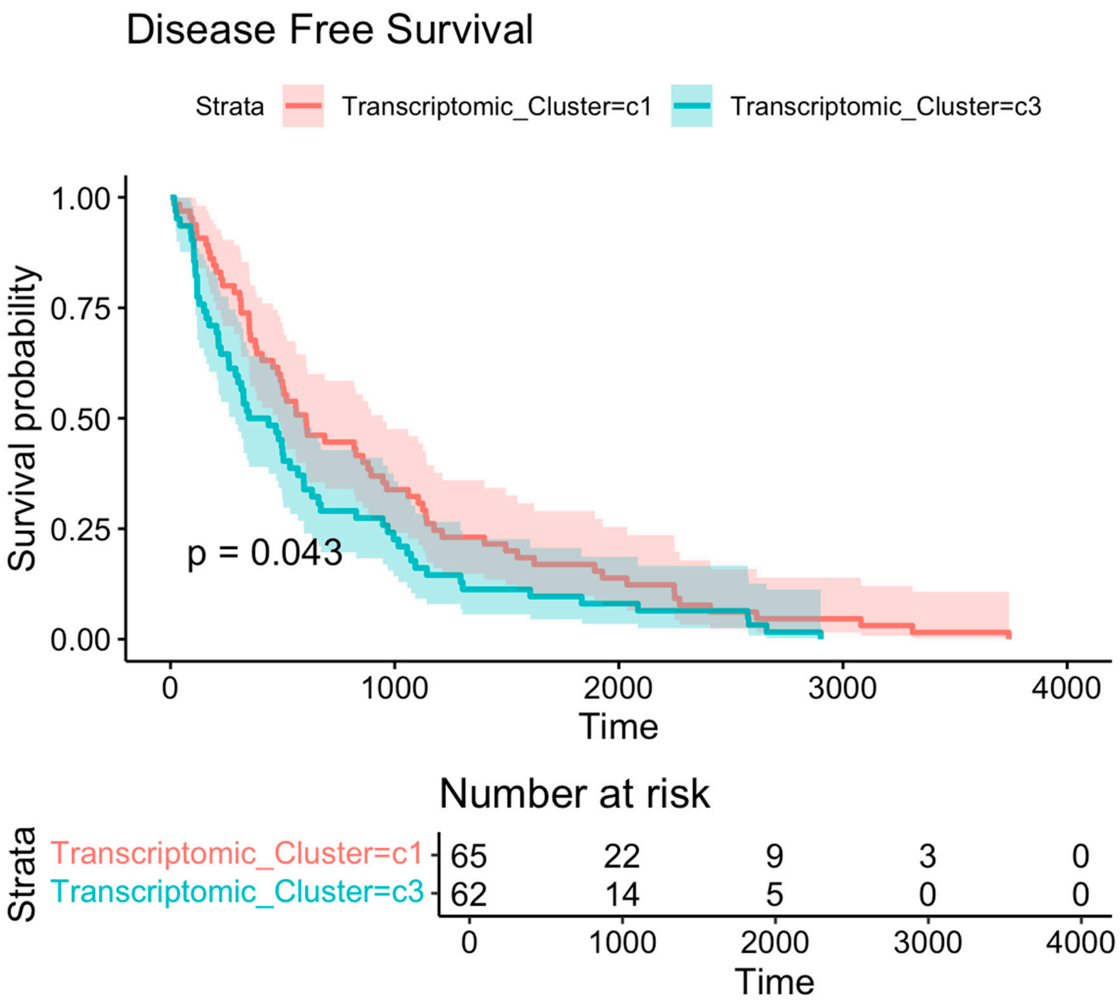

Figure S6. Survival analysis (using the Kaplan-Meier method) of the TCGA-SARC (external cohort) patients classified per transcriptomic cluster/subtype (C1 and C3): DFS analysis by the Kaplan-Meier method and respective curves.

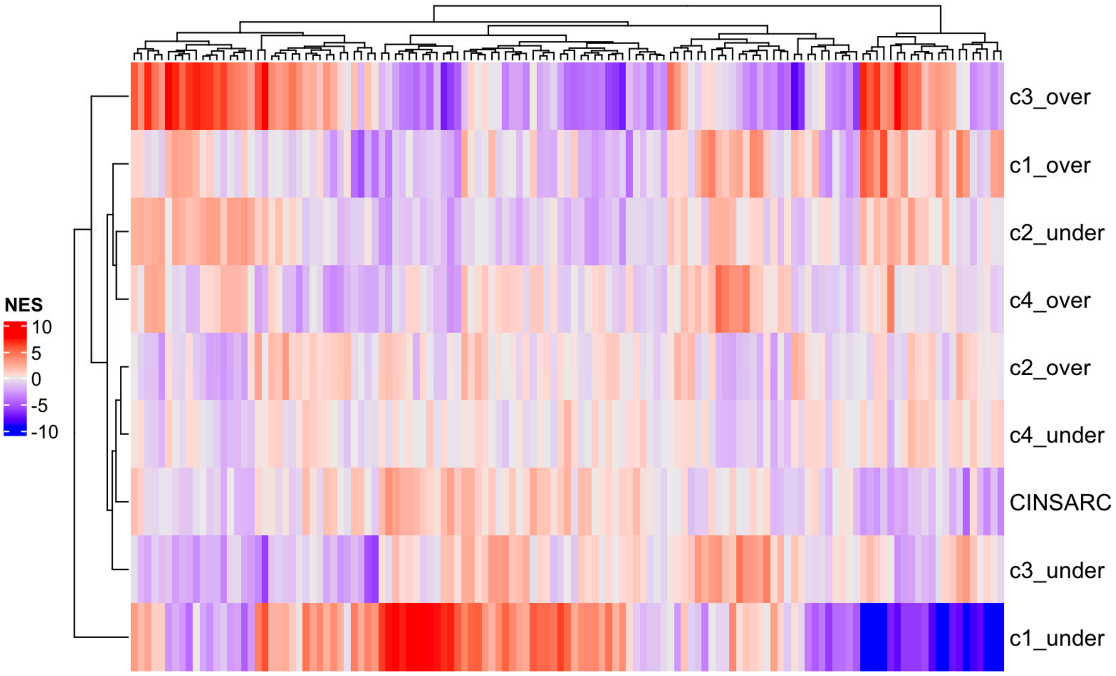

Figure S7. Persistence of ssGSEA enrichment in C1_under and C3_over of the TCGA-SARC population persist following the exclusion of UPS patients.

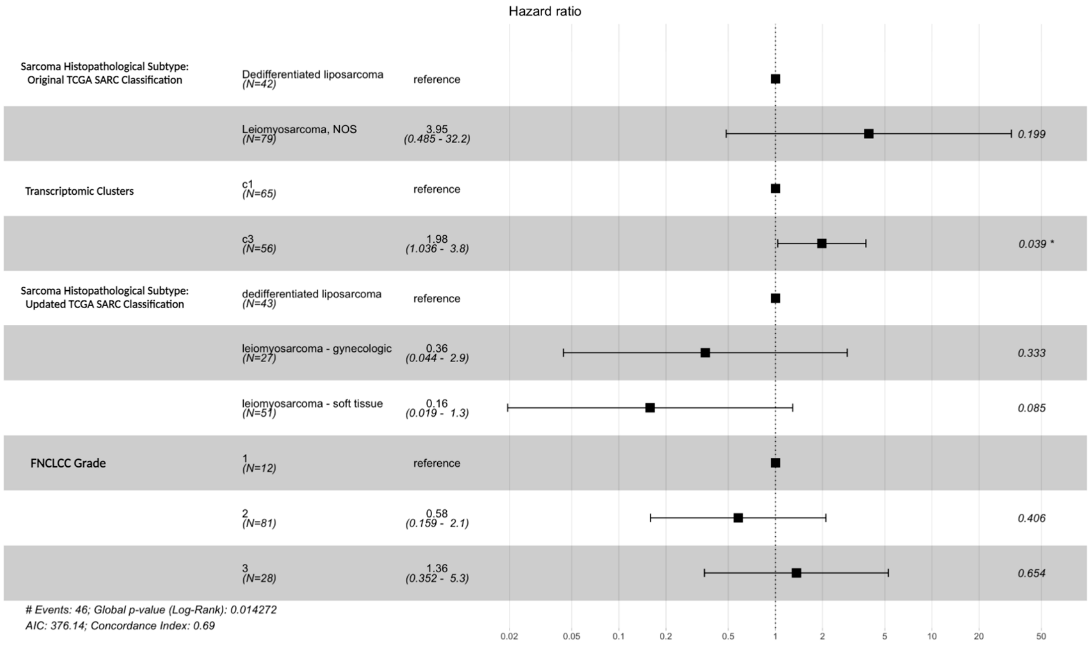

Figure S8. Differential impact of distinct histopathological and molecular factors on overall survival considering TCGA-SARC patients, after the exclusion of UPS patients (Forest plot).

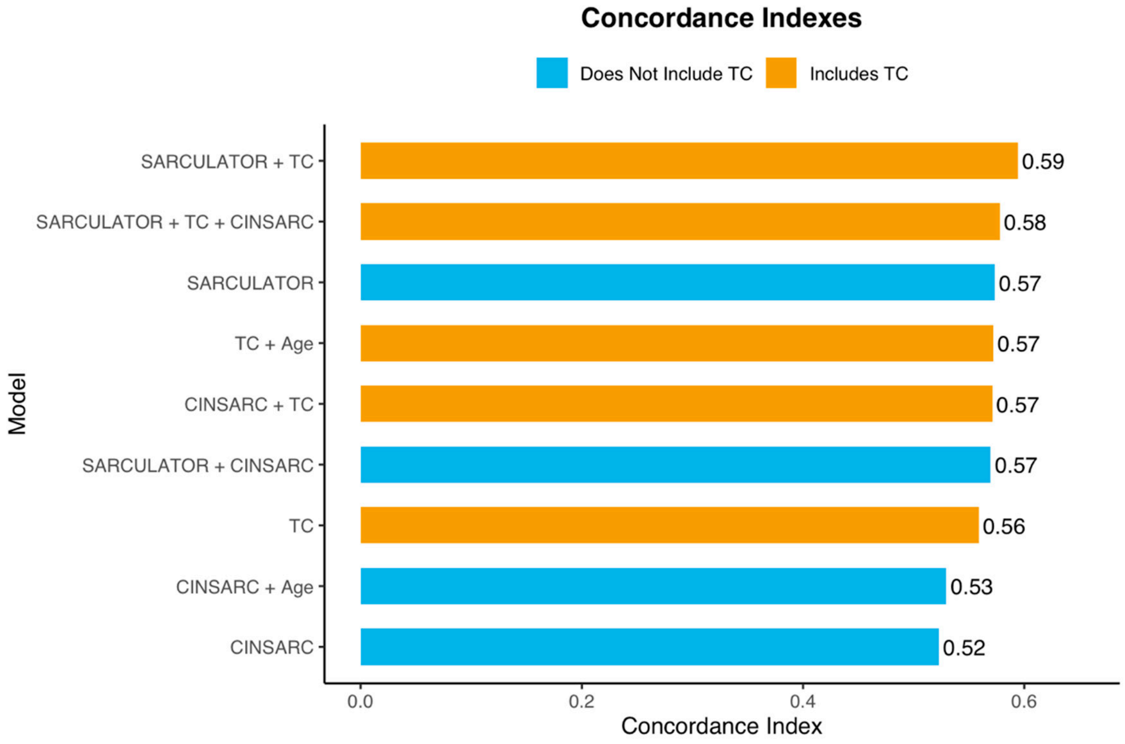

Figure S9. Transcriptomic cluster-based classification, and other clinical and molecular-based models' prognostic performance in terms of DFS. Bar chart showing the concordance indexes of different prognostic models employed using the population of the validation cohort (TCGA-SARC) (including CINSARC, CINSARC + Age, SARC, TC, TC + Age, SARC + CINSARC, SARC + TC, SARC + TC + CINSARC).

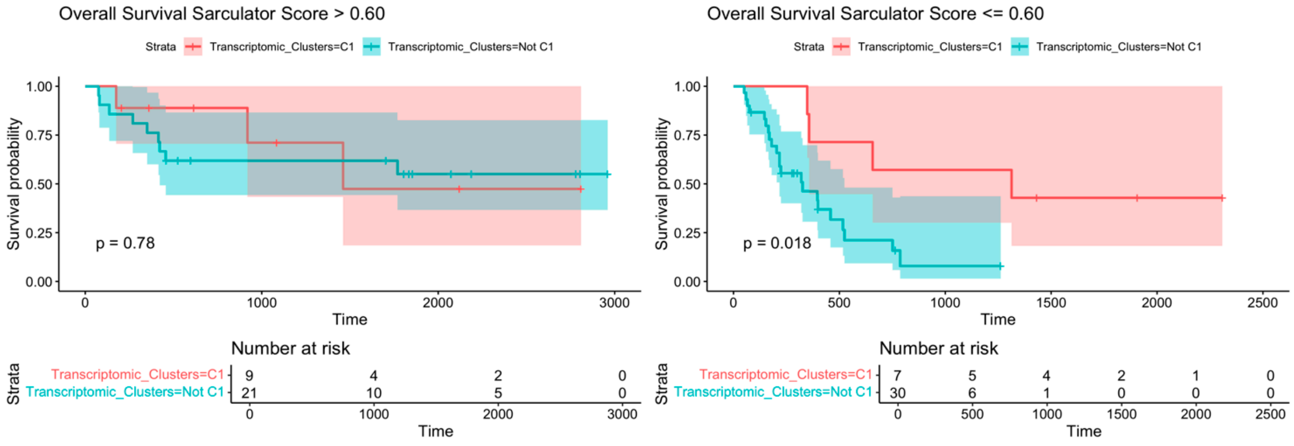

Figure S10. Survival analysis (using the Kaplan-Meier method) of the study cohort patients that have displayed either a 5-year OS ≤ 60% and a 5-year OS > 60% (Sarculator-based): OS analysis by the Kaplan-Meier method and respective curves.

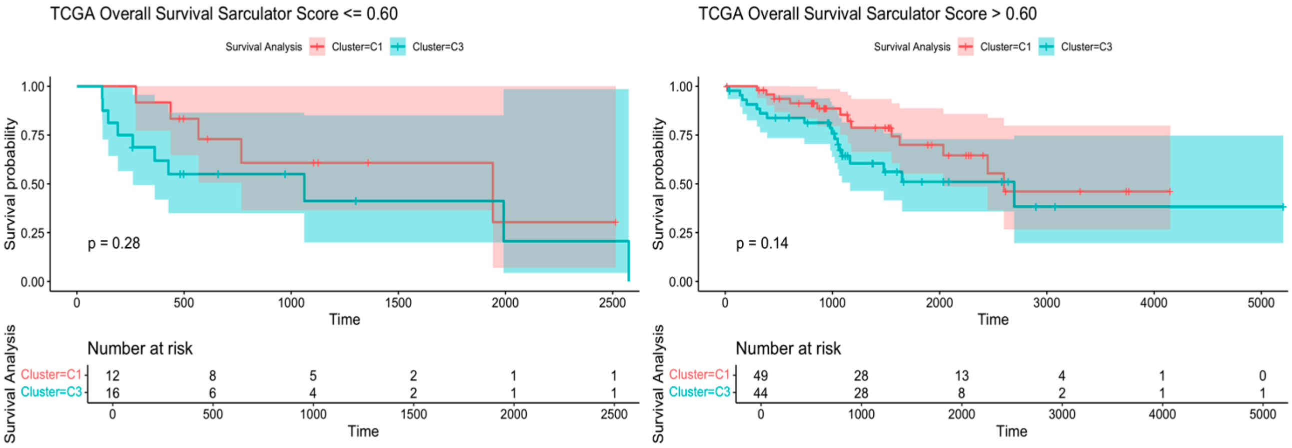

(A)

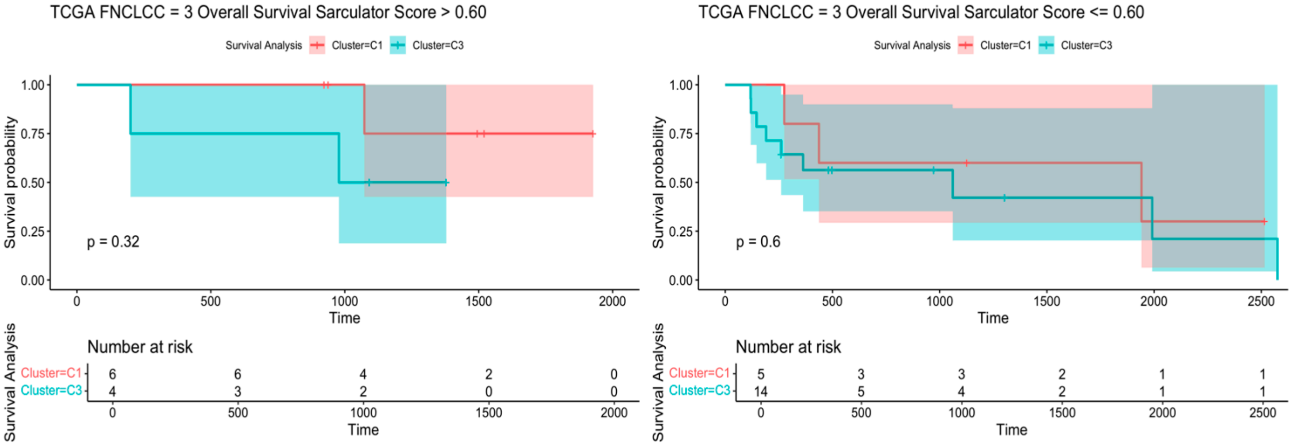

(B)

Figure S11. Survival analysis (using the Kaplan-Meier method) of the TCGA-SARC (external cohort) patients that have displayed either a 5-year OS ≤ 60% and a 5-year OS > 60% (Sarculator-based): OS analysis by the Kaplan-Meier method and respective curves. The generic analysis including DDLPS, LMS and UPS patients from the TCGA-SARC cohort is displayed on **Supplementary Figure 11A**. The specific analysis including DDLPS, LMS and UPS patients from the TCGA-SARC cohort and that display an STS with an FNLCC grade 3 are shown on **Supplementary Figure 11B.**

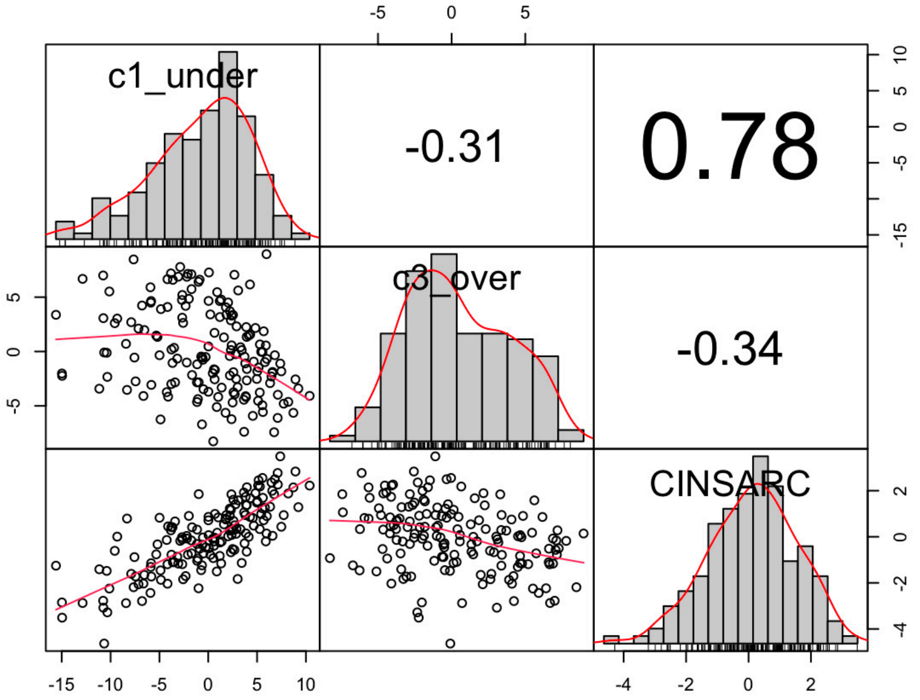

Figure S12. Correlation between ssGSEA enrichment scores (NES) from c1_under, c3_over and CINSARC genes (Spearman correlation plot).

Table S1. Demographic characteristics of the study population and main features of the included STS cases.

	Total (n=101)
**Age at diagnosis**, median [IQR], years	67 [19.7]
**Gender**, n (%)	
Male	50 (49.5)
Female	51 (50.5)
**Histopathological subtypes**, n (%)	
Dedifferentiated liposarcoma	25 (24.8)
Leiomyosarcoma	25 (24.8)
Undifferentiated pleomorphic sarcoma	51 (50.4)
**Location**, n (%)	
Upper limb	9 (8.9)
Lower limb	49 (48.5)
Retroperitoneum	31 (30.7)
Trunk	12 (11.9)
**Presentation type**, n (%)	
Localized	96 (95.0)
Distant metastasis	5 (5.0)
**Size of the primary tumor**, median [IQR], cm	13 [10.0]
Missing	4

Table S2. Surgical and systemic treatment details of the patients who were considered for a curative surgical approach at IPOLFG.

	Total (n=94)
**Neoadjuvant treatment,** n (%), n=94	
No neoadjuvant treatment	91 (96.8)
Neoadjuvant treatment	3 (3.2)
**Resectability,** n (%), n = 94	
Resectable	92 (97.9)
Unresectable	2 (2.1)
**Resection margins,** n (%), n=92	
R0/R1	89 (96.7)
R2	3 (3.3)
**Adjuvant treatment,** n (%), n=92	
Radiotherapy	55 (59.7)
No adjuvant treatment	34 (37.0)
Chemotherapy or Chemoradiotherapy	3 (3.3)

Table S3. Actionable gene variants (distributed per ESCAT evidence tier) found for patients included in C1. For each variant, gene identification, nature of the alteration, functional relevance evidence for the alteration (A – Curated; B – Assumed; C - Predicted) and the predictive value of the alteration is provided (2 - Investigational; 3 – Hypothetical target: Alteration-drug match is associated with antitumor activity, but magnitude of benefit is unknown (potential cancer-repurposing opportunity); 4 – Hypothetical target: pre-clinical evidence of actionability).

Tier	Gene	Alteration	Functional relevance evidence	Biomarker predictive value
2	MDM 2	Copy number alteration (Amplification)	Evidence A<br>Oncogenic	Sensitivity/Response:<br>- Brigimadlin.<br>- Milademetan.
	MTAP	Copy number alteration (Deletion)	Evidence A	Sensitivity/Response:

			Likely oncogenic	- MRTX1719.<br>- AMG193.
	TP53	Mutation – missense p.Tyr234Asp exon 7/11	Evidence A<br>Pathogenic / Likely Pathogenic	Sensitivity/Response:<br>- Pazopanib.<br>- Vorinostat.
	TP53	Mutation – frameshift p.Glu339Ter exon 10/11	Evidence A<br>Pathogenic	Sensitivity/Response:<br>- Pazopanib.<br>- Vorinostat.
	TP53	Mutation - stop gained p.Arg196Ter exon 6/11	Evidence A<br>Pathogenic	Sensitivity/Response:<br>- Pazopanib.<br>- Vorinostat.
	TP53	Mutation – missense p.Met246Lys exon 7/11	Evidence A<br>Likely Pathogenic	Sensitivity/Response:<br>- Pazopanib.<br>- Vorinostat.
**3**	MDM2	Copy number alteration (Amplification)	Evidence A<br>Oncogenic	Sensitivity/Response:<br>- Brigimadlin.<br>- Ezabenlimab.
	NF1	Mutation - stop gained p.Gln1870Ter exon 38/57	Evidence A<br>Pathogenic	Sensitivity/Response:<br>- Selumetinib.<br>Resistance/Reduced Sensitivity:<br>- Vemurafenib.
	TP53	Mutation – missense p.Tyr234Asp exon 7/11	Evidence A<br>Pathogenic / Likely Pathogenic<br>Likely Oncogenic	Sensitivity/Response:<br>- Azacitidne.<br>- Eprenetapopt.<br>- Chemotherapy.<br>Resistance/Reduced Sensitivity:<br>- RG7112.
	TP53	Mutation – frameshift p.Glu339Ter exon 10/11	Evidence A<br>Pathogenic	Sensitivity/Response:<br>- Azacitidne.<br>- Eprenetapopt.<br>- Chemotherapy.<br>Resistance/Reduced Sensitivity:<br>- RG7112.
	TP53	Mutation - stop gained p.Arg196Ter exon 6/11	Evidence A<br>Pathogenic	Sensitivity/Response:<br>- Azacitidne.<br>- Eprenetapopt.<br>- Chemotherapy.<br>Resistance/Reduced Sensitivity:<br>- RG7112.
	TP53	Mutation – missense p.Met246Lys exon 7/11	Evidence A<br>Likely Pathogenic<br>Likely Oncogenic	Sensitivity/Response:<br>- Azacitidne.<br>- Eprenetapopt.<br>- Chemotherapy.<br>Resistance/Reduced Sensitivity:<br>- RG7112.
	RAD51B	Mutation – frameshift p.Leu31PhefsTer9 exon 3/11	Evidence B	Sensitivity/Response:<br>- Olaparib.
	NF1	Mutation – frameshift p.Gln2702HisfsTer6 exon 56/57	Evidence B	Sensitivity/Response:<br>- Selumetinib.<br>Resistance/Reduced Sensitivity:<br>- Vemurafenib.

	ATM	Mutation – missense p.Ser2408Leu exon 49/63	Evidence C	Sensitivity/Response:<br>- Olaparib.<br>- Talazoparib.
	BRIP1	Mutation – missense p.Arg264Trp exon 7/20	Evidence C	Sensitivity/Response:<br>- Olaparib.
4	CDK4	Copy number alteration (Amplification)	Evidence A Oncogenic	Sensitivity/Response:<br>- Palbociclib.
	MDM2	Copy number alteration (Amplification)	Evidence A Oncogenic	Sensitivity/Response:<br>- Brigimadlin.<br>- Milademetan.
	TP53	Mutation - stop gained p.Arg196Ter exon 6/11	Evidence A Pathogenic	Resistance/Reduced Sensitivity:<br>- Rebemadlin.<br>- MDM2 inhibitor AMGMDS3.
	TP53	Mutation – missense p.Met246Lys exon 7/11	Evidence A Likely pathogenic Likely oncogenic	Resistance/Reduced Sensitivity:<br>- Rebemadlin.<br>- MDM2 inhibitor AMGMDS3.
	TP53	Mutation – missense p.Tyr234Asp exon 7/11	Evidence A Likely pathogenic Likely oncogenic	Resistance/Reduced Sensitivity:<br>- Rebemadlin.<br>- MDM2 inhibitor AMGMDS3.
	TP53	Mutation – frameshift p.Glu339Ter exon 10/11	Evidence A Pathogenic	Resistance/Reduced Sensitivity:<br>- Rebemadlin.<br>- MDM2 inhibitor AMGMDS3.
	NF1	Mutation - stop gained p.Gln1870Ter exon 38/57.	Evidence A Pathogenic	Sensitivity/Response:<br>- Trametinib.<br>- Cobimetinib.
	NF1	Mutation – frameshift p.Gln2702HisfsTer6 exon 56/57	Evidence B	Sensitivity/Response:<br>- Trametinib.<br>- Cobimetinib

Note: It is important to underline an important number of alterations without a specific predictive value, but with clear functional relevance evidence of the genes AMER1, NTRK1, PTEN, PI3KCA, RB1.

Table S4. Actionable gene variants (distributed per ESCAT evidence tier) found for patients included in C2. For each variant, gene identification, nature of the alteration, functional relevance evidence for the alteration (A – Curated; B – Assumed; C - Predicted) and the predictive value of the alteration is provided (2 - Investigational; 3 – Hypothetical target: Alteration-drug match is associated with antitumor activity, but magnitude of benefit is unknown (potential cancer-repurposing opportunity); 4 – Hypothetical target: pre-clinical evidence of actionability).

Tier	Gene	Alteration	Functional relevance evidence	Biomarker predictive value
	MDM 2	Copy number alteration (Amplification)	Evidence A Oncogenic	Sensitivity/Response:<br>- Brigimadlin.<br>- Milademetan.
2	TP53	Mutation – missense p.Val157Phe exon 5/11	Evidence A Likely oncogenic Resistance	Sensitivity/Response:<br>- Pazopanib.<br>- Vorinostat.
	TP53	Mutation - stop gained p.Gln144Ter exon 5/11	Evidence A Pathogenic	Sensitivity/Response:<br>- Pazopanib.

				- Vorinostat.
	TP53	Mutation – missense p.Ile195Thr exon 6/11	Evidence A Likely oncogenic	Sensitivity/Response:<br>- Pazopanib.<br>- Vorinostat.
	TP53	Mutation – frameshift p.Arg209LysfsTer6 exon 6/11	Evidence A Pathogenic	Sensitivity/Response:<br>- Pazopanib.<br>- Vorinostat.
	TP53	Mutation – missense p.Tyr126Asn exon 5/11	Evidence A Likely oncogenic	Sensitivity/Response:<br>- Pazopanib.<br>- Vorinostat.
	TP53	Mutation - stop gained p.Arg306Ter exon 8/11	Evidence A Pathogenic	Sensitivity/Response:<br>- Pazopanib.<br>- Vorinostat.
	TP53	Mutation - stop gained p.Gln144Ter exon 5/11	Evidence A Pathogenic	Sensitivity/Response:<br>- Pazopanib.<br>- Vorinostat.
	MTAP	Copy number alteration (Deletion)	Evidence A Likely oncogenic	Sensitivity/Response:<br>- MRTX1719.<br>- AMMG193.
	ERBB2	Copy number alteration (Amplification)	Evidence A Oncogenic	Sensitivity/Response:<br>- Trastuzumab Deruxtecan.
	TP53	Mutation – frameshift p.His178ProfsTer69 exon 5/11	Evidence B	Sensitivity/Response:<br>- Pazopanib.<br>- Vorinostat.
	TP53	Mutation - stop gained p.Cys182Ter exon 5/11	Evidence B	Sensitivity/Response:<br>- Pazopanib.<br>- Vorinostat.
	TSC2	Mutation – frameshift p.Gly654ValfsTer2 exon 19/42	Evidence B	Sensitivity/Response:<br>- ABI-009.
	TSC2	Mutation – missense p.Arg59Gln exon 3/42	Evidence C	Sensitivity/Response:<br>- ABI-009.
**3**	FGFR1	Mutation – missense p.Asn577Lys exon 13/19	Evidence A Pathogenic Likely oncogenic Sensitivity/Response	Sensitivity/Response:<br>- Pemigatinib.
	MDM2	Copy number alteration (amplification)	Evidence A Oncogenic	Sensitivity/Response:<br>- Brigimadlin.<br>- Ezabenlimab.
	RET	Mutation – missense p.Val648Ile exon 11/20	Evidence A Likely oncogenic	Sensitivity/Response:<br>- Selpercatinib.<br>- Pralsetinib.
	ERBB2	Copy number alteration (amplification)	Evidence A Oncogenic	Sensitivity/Response:<br>- Trastuzumab.<br>- Trastuzumab, Pertuzumab.<br>- Trastuzumab, Chemotherapy.<br>- Ado-Trastuzumab Emtansine.<br>- Trastuzumab Deruxtecan.<br>- Tucatinib, Trastuzumab.

			- Tucatinib, Trastuzumab, Capecitabine.<br>- Lapatinib, Capecitabine.<br>- Lapatinib, Trastuzumab.<br>- Neratinib.<br>- Neratinib, Capecitabine.<br>- Margetuximab, Chemotherapy.
NF1	Mutation - stop gained p.Trp571Ter exon 15/57	Evidence A Pathogenic	Sensitivity/Response:<br>- Selumetinib.<br>Resistance/Reduced Sensitivity:<br>- Vemurafenib.
POLE	Mutation – missense p.Gly6Arg exon 1/49	Evidence A Likely oncogenic	Sensitivity/Response:<br>- Pembrolizumab.
TP53	Mutation – missense p.Val157Phe exon 5/11	Evidence A Likely oncogenic Resistance	Sensitivity/Response:<br>- Azacitidne.<br>- Eprenetapopt.<br>- Chemotherapy.<br>Resistance/Reduced Sensitivity:<br>- RG7112.
TP53	Mutation - stop gained p.Gln144Ter exon 5/11	Evidence A Pathogenic	Sensitivity/Response:<br>- Azacitidne.<br>- Eprenetapopt.<br>- Chemotherapy.<br>Resistance/Reduced Sensitivity:<br>- RG7112.
TP53	Mutation – missense p.Ile195Thr exon 6/11	Evidence A Likely oncogenic	Sensitivity/Response:<br>- Azacitidne.<br>- Eprenetapopt.<br>- Chemotherapy.<br>Resistance/Reduced Sensitivity:<br>- RG7112.
TP53	Mutation – missense p.Tyr126Asn exon 5/11	Evidence A Likely oncogenic	Sensitivity/Response:<br>- Azacitidne.<br>- Eprenetapopt.<br>- Chemotherapy.<br>Resistance/Reduced Sensitivity:<br>- RG7112.
TP53	Mutation - stop gained p.Arg306Ter exon 8/11	Evidence A Pathogenic	Sensitivity/Response:<br>- Azacitidne.<br>- Eprenetapopt.<br>- Chemotherapy.<br>Resistance/Reduced Sensitivity:<br>- RG7112.
TP53	Mutation - stop gained p.Gln144Ter exon 5/11	Evidence A Pathogenic	Sensitivity/Response:<br>- Azacitidne.<br>- Eprenetapopt.<br>- Chemotherapy.<br>Resistance/Reduced Sensitivity:<br>- RG7112.
PTEN	Mutation – frameshift p.Ile101LysfsTer5 exon 5/9	Evidence B	Sensitivity/Response:<br>- Fulvestrant.

				- Capivasertib.
	PTEN	Mutation – frameshift p.Glu40ValfsTer20 exon 2/9	Evidence B	Sensitivity/Response: - Fulvestrant. - Capivasertib.
4	TP53	Mutation – frameshift p.His178ProfsTer69 exon 5/11	Evidence B	Sensitivity/Response: - Azacitidne. - Eprenetapopt. - Chemotherapy. Resistance/Reduced Sensitivity: - RG7112.
	TP53	Mutation - stop gained p.Cys182Ter exon 5/11	Evidence B	Sensitivity/Response: - Azacitidne. - Eprenetapopt. - Chemotherapy. Resistance/Reduced Sensitivity: - RG7112.
	RAD51C	Mutation – frameshift p.Val298AlafsTer6 exon 6/9	Evidence B	Sensitivity/Response: - Olaparib. - Talazoparib.
	TSC2	Mutation – frameshift p.Gly654ValfsTer2 exon 19/42	Evidence B	Sensitivity/Response: - Everolimus. - ABI-009.
	TSC2	Mutation – missense p.Arg59Gln exon 3/42	Evidence C	Sensitivity/Response: - Everolimus. - ABI-009.
	CDK4	Copy number alteration (Amplification)	Evidence A Oncogenic	Sensitivity/Response: - Palbociclib. - Abemaciclib.
	FGFR1	Mutation – missense p.Asn577Lys exon 13/19	Evidence A Pathogenic Likely oncogenic Sensitivity/Response	Sensitivity/Response: - AZD4547. - Erdafitinib.
	MDM2	Copy number alteration (Amplificaton)	Evidence A Oncogenic.	Sensitivity/Response: - Brigimadlin. - Milademetan.
	TP53	Mutation – missense p.Val157Phe exon 5/11	Evidence A Likely oncogenic. Resistance.	Resistance/Reduced Sensitivity: - Rebemadlin. - MDM2 inhibitor AMGMDS3.
	TP53	Mutation - stop gained p.Gln144Ter exon 5/11	Evidence A Pathogenic.	Resistance/Reduced Sensitivity: - Rebemadlin. - MDM2 inhibitor AMGMDS3.
	TP53	Mutation – missense p.Ile195Thr exon 6/11	Evidence A Likely oncogenic	Resistance/Reduced Sensitivity: - Rebemadlin. - MDM2 inhibitor AMGMDS3.
	TP53	Mutation – frameshift p.Arg209LysfsTer6 exon 6/11	Evidence A Pathogenic	Resistance/Reduced Sensitivity: - Rebemadlin. - MDM2 inhibitor AMGMDS3.

	TP53	Mutation – missense<br>p.Tyr126Asn exon 5/11	Evidence A<br>Likely oncogenic.	Resistance/Reduced Sensitivity:<br>- Rebemadlin.<br>- MDM2 inhibitor AMGMDS3.
	TP53	Mutation - stop gained<br>p.Arg306Ter exon 8/11	Evidence A<br>Pathogenic.	Resistance/Reduced Sensitivity:<br>- Rebemadlin.<br>- MDM2 inhibitor AMGMDS3.
	TP53	Mutation - stop gained<br>p.Gln144Ter exon 5/11	Evidence A<br>Pathogenic	Resistance/Reduced Sensitivity:<br>- Rebemadlin.<br>- MDM2 inhibitor AMGMDS3.
	CCNE1	Copy number alteration<br>(Amplification)	Evidence A<br>Oncogenic.	Sensitivity/Response:<br>- RP-6306.
	NF1	Mutation - stop gained<br>p.Trp571Ter exon 15/5	Evidence A<br>Pathogenic.	Sensitivity/Response:<br>- Trametinib.<br>- Cobimetinib.
	PTEN	Mutation – frameshift<br>p.Glu40ValfsTer20 exon 2/9	Evidence B	Sensitivity/Response:<br>- GSK2636771.<br>- AZD8186.<br>- Ipatasertib.
	PTEN	Mutation – frameshift<br>p.Ile101LysfsTer5 exon 5/9	Evidence B	Sensitivity/Response:<br>- GSK2636771.<br>- AZD8186.<br>- Ipatasertib.
	TP53	Mutation – frameshift<br>p.His178ProfsTer69 exon 5/11	Evidence B	Resistance/Reduced Sensitivity:<br>- Rebemadlin.<br>- MDM2 inhibitor AMGMDS3.
	TP53	Mutation - stop gained<br>p.Cys182Ter<br>exon 5/11	Evidence B	Resistance/Reduced Sensitivity:<br>- Rebemadlin.<br>- MDM2 inhibitor AMGMDS3.

Note: It is important to underline an important number of alterations without a specific predictive value, but with clear functional relevance evidence of the genes IGF1R, MUTYH, AURKA, AURKB, BRD4, NTRK1, VEGF, HGF.

Table S5. Actionable gene variants (distributed per ESCAT evidence tier) found for patients included in C3. For each variant, gene identification, nature of the alteration, functional relevance evidence for the alteration (A – Curated; B – Assumed; C - Predicted) and the predictive value of the alteration is provided (2 - Investigational; 3 – Hypothetical target: Alteration-drug match is associated with antitumor activity, but magnitude of benefit is unknown (potential cancer-repurposing opportunity); 4 – Hypothetical target: pre-clinical evidence of actionability).

Tier	Gene	Alteration	Functional relevance evidence	Biomarker predictive value
**2**	MDM 2	Copy number alteration<br>(Amplification)	Evidence A<br>Oncogenic	Sensitivity/Response:<br>- Brigimadlin.<br>- Milademetan.
	MTAP	Copy number alteration<br>(Deletion)	Evidence A<br>Likely oncogenic	Sensitivity/Response:<br>- MRTX1719.<br>- AMG193.
	TP53	Mutation – missense<br>p.His193Tyr exon 6/11	Evidence A	Sensitivity/Response:<br>- Pazopanib.

			Pathogenic / Likely Pathogenic<br>Likely oncogenic	- Vorinostat.
	TP53	Mutation – missense<br>p.Ala159Val exon 5/11	Evidence A<br>Pathogenic<br>Likely pathogenic<br>Likely oncogenic	Sensitivity/Response:<br>- Pazopanib.<br>- Vorinostat.
	TP53	Mutation – missense<br>p.Val173Leu exon 5/11	Evidence A<br>Pathogenic<br>Likely pathogenic<br>Likely oncogenic	Sensitivity/Response:<br>- Pazopanib.<br>- Vorinostat.
	TP53	Mutation – missense<br>p.Arg175His exon 5/1	Evidence A<br>Pathogenic<br>Oncogenic<br>Poor outcome	Sensitivity/Response:<br>- Pazopanib.<br>- Vorinostat.
	TP53	Mutation – missense<br>p.Arg181Cys exon 5/1	Evidence A<br>Likely oncogenic	Sensitivity/Response:<br>- Pazopanib.<br>- Vorinostat.
	TP53	Mutation – missense<br>p.Arg248Trp exon 7/1	Evidence A<br>Pathogenic<br>Likely oncogenic	Sensitivity/Response:<br>- Pazopanib.<br>- Vorinostat.
	TP53	Mutation – missense<br>p.Arg273Cys exon 8/11	Evidence A<br>Pathogenic<br>Likely pathogenic	Sensitivity/Response:<br>- Pazopanib.<br>- Vorinostat.
	TP53	Mutation – missense<br>p.Arg282Gly exon 8/11	Evidence A<br>Pathogenic<br>Likely oncogenic	Sensitivity/Response:<br>- Pazopanib.<br>- Vorinostat.
	TP53	Mutation – missense<br>p.Glu286Lys exon 8/11	Evidence A<br>Pathogenic<br>Likely pathogenic<br>Likely oncogenic	Sensitivity/Response:<br>- Pazopanib.<br>- Vorinostat.
	TSC2	Mutation – missense<br>p.Arg1713His exon 40/42	Evidence A<br>Pathogenic<br>Likely pathogenic	Sensitivity/Response:<br>- ABI-009.
	PIK3CA	Mutation – missense<br>p.Ala1066Val exon 21/21	Evidence A<br>Likely oncogenic	Sensitivity/Response:<br>- Capivasertib.<br>- Copanlisib.
**3**	MDM2	Copy number alteration<br>(Amplification)	Evidence A<br>Oncogenic	Sensitivity/Response:<br>- Brigimadlin.<br>- Ezabenlimab.
	POLE	Mutation – missense<br>p.Gly6Arg exon 1/49	Evidence A<br>Likely oncogenic	Sensitivity/Response:<br>- Pembrolizumab.
	POLE	Mutation – missense<br>p.Gly6Arg exon 1/49	Evidence A<br>Likely oncogenic	Sensitivity/Response:<br>- Pembrolizumab.
	TSC2	Mutation – missense<br>p.Arg1713His exon 40/42	Evidence A<br>Pathogenic<br>Likely pathogenic	Sensitivity/Response:<br>- Everolimus.<br>- ABI-009.
	ATM	Mutation – missense<br>p.Arg337His exon 8/63	Evidence A<br>Likely oncogenic	Sensitivity/Response:<br>- Olaparib.<br>- Talazoparib.

	KRAS	Mutation – missense<br>p.Thr20Ala exon 2/5	Evidence A<br>Resistance<br>Poor outcome	Sensitivity/Response:<br>- Cobimetinib.<br>- Trametinib.<br>- Binimetinib.<br>- Pemetrexed, Trametinib, Docetaxel.<br>- Docetaxel, Selumetinib.<br>- Abemaciclib.<br>- Nivolumab, Atezolizumab.<br>Resistance/Reduced Sensitivity:<br>- Panitumumab.<br>- Tucatinib, Trastuzumab.<br>- Cetuximab.<br>- Panitumumab.<br>- Gemcitabine, Erlotinib.<br>- Gefitinib.<br>- Bevacizumab.
	KRAS	Mutation – missense<br>p.Leu19Phe exon 2/5	Evidence A<br>Oncogenic<br>Resistance<br>Poor outcome	Sensitivity/Response:<br>- Cobimetinib.<br>- Trametinib.<br>- Binimetinib.<br>- Docetaxel, Selumetinib.<br>- Pemetrexed, Trametinib, Docetaxel.<br>- Abemaciclib.<br>- Nivolumab, Atezolizumab.<br>Resistance/Reduced Sensitivity:<br>- Panitumumab.<br>- Tucatinib, Trastuzumab.<br>- Cetuximab.<br>- Panitumumab.<br>- Gemcitabine, Erlotinib.<br>- Gefitinib.<br>- Bevacizumab.
	NRAS	Mutation – missense<br>p.Gln61Lys exon 3/7	Evidence A<br>Oncogenic<br>Resistance	Sensitivity/Response:<br>- Cobimetinib.<br>- Trametinib.<br>- Binimetinib.<br>- Selumetinib.<br>Resistance/Reduced Sensitivity:<br>- Panitumumab.<br>- Tucatinib, Trastuzumab.<br>- Cetuximab.<br>- Panitumumab.<br>- Vemurafenib.
	MET	Copy number alteration<br>(Amplification)	Evidence A<br>Oncogenic	Sensitivity/Response:<br>- Telisotuzumab Vedotin.<br>- Tepotinib.<br>- Capmatinib.<br>- Crizotinib.<br>Resistance/Reduced Sensitivity:<br>- Osimertinib.<br>- Gefitinib.

			- Erlotinib.
PIK3CA	Mutation - missense<br>p.Ala1066Val<br>exon 21/21	Evidence A<br>Likely oncogenic	Sensitivity/Response:<br>- Capivasertib, Fulvestrant.<br>- Alpelisib, Fulvestrant.<br>- Everolimus.<br>Resistance/Reduced Sensitivity:<br>- Trastuzumab.<br>- Lapatinib, Capecitabine.<br>- Cetuximab.<br>- Panitumumab.<br>- Erlotinib.<br>- Gefitinib.
PTEN	Mutation – frameshift<br>p.Lys267ArgfsTer9 exon 7/9	Evidence A<br>Pathogenic.	Sensitivity/Response:<br>- Capivasertib, Fulvestrant.
TP53	Mutation – missense<br>p.His193Tyr exon 6/11	Evidence A<br>Pathogenic<br>Likely pathogenic<br>Likely oncogenic	Sensitivity/Response:<br>- Azacytidine.<br>- Eprenetapopt.<br>- Chemotherapy.<br>Resistance/Reduced Sensitivity:<br>- RG7112.
TP53	Mutation – missense<br>p.Val173Leu exon 5/11	Evidence A<br>Pathogenic<br>Likely pathogenic<br>Likely oncogenic	Sensitivity/Response:<br>- Azacytidine.<br>- Eprenetapopt.<br>- Chemotherapy.<br>Resistance/Reduced Sensitivity:<br>- RG7112.
TP53	Mutation – missense<br>p.Arg175His exon 5/11	Evidence A<br>Pathogenic<br>Oncogenic	Sensitivity/Response:<br>- Azacytidine.<br>- Eprenetapopt.<br>- Chemotherapy.<br>Resistance/Reduced Sensitivity:<br>- RG7112.
TP53	Mutation – missense<br>p.Arg181Cys exon 5/11	Evidence A<br>Likely oncogenic	Sensitivity/Response:<br>- Azacytidine.<br>- Eprenetapopt.<br>- Chemotherapy.<br>Resistance/Reduced Sensitivity:<br>- RG7112.
TP53	Mutation – missense<br>p.Arg248Trp exon 7/11	Evidence A<br>Pathogenic<br>Likely oncogenic	Sensitivity/Response:<br>- Azacytidine.<br>- Eprenetapopt.<br>- Chemotherapy.<br>Resistance/Reduced Sensitivity:<br>- RG7112.
TP53	Mutation – missense<br>p.Arg273Cys exon 8/11	Evidence A<br>Pathogenic<br>Likely pathogenic<br>Likely oncogenic	Sensitivity/Response:<br>- Azacytidine.<br>- Eprenetapopt.<br>- Chemotherapy.<br>Resistance/Reduced Sensitivity:<br>- RG7112.

	TP53	Mutation – missense<br>p.Arg282Gly exon 8/11	Evidence A<br>Pathogenic<br>Likely oncogenic	Sensitivity/Response:<br>- Azacytidine.<br>- Eprenetapopt.<br>- Chemotherapy.<br>Resistance/Reduced Sensitivity:<br>- RG7112.
	TP53	Mutation – missense<br>p.Glu286Lys exon 8/11	Evidence A<br>Pathogenic<br>Likely pathogenic<br>Likely oncogenic	Sensitivity/Response:<br>- Azacytidine.<br>- Eprenetapopt.<br>- Chemotherapy.<br>Resistance/Reduced Sensitivity:<br>- RG7112.
	NF1	Mutation – frameshift<br>p.Tyr930PhefsTer8 exon 21/57	Evidence B	Sensitivity/Response:<br>- Selumetinib.<br>Resistance/Reduced Sensitivity:<br>- Vemurafenib.
	ATM	Mutation – missense<br>p.Arg2854Cys exon 58/63	Evidence C	Sensitivity/Response:<br>- Olaparib.<br>- Talazoparib.
	ATR	Mutation – missense<br>p.Ser1154Thr exon 18/47	Evidence C	Sensitivity/Response:<br>- Talazoparib.
	VHL	Mutation – missense<br>p.Lys196Glu exon 3/3	Evidence C	Sensitivity/Response:<br>- Everolimus.
**4**	CDK4	Copy number alteration<br>(Amplification)	Evidence A<br>Oncogenic	Sensitivity/Response:<br>- Abemaciclib.<br>- Palbociclib.
	MDM2	Copy number alteration<br>(Amplification)	Evidence A<br>Oncogenic	Sensitivity/Response:<br>- Brigimadlin.<br>- Milademetan.
	CDKN2A	Mutation - stop gained<br>p.Arg58Ter exon 2/3	Evidence A<br>Pathogenic	Sensitivity/Response:<br>- Abemaciclib.<br>- Palbociclib.<br>- Ribociclib.
	TP53	Mutation – missense<br>p.His193Tyr exon 6/11	Evidence A<br>Pathogenic<br>Likely pathogenic<br>Likely oncogenic	Resistance/Reduced Sensitivity:<br>- Rebemadlin.<br>- MDM2 inhibitor AMGMDS2.
	TP53	Mutation – missense<br>p.Ala159Val exon 5/11	Evidence A<br>Pathogenic<br>Likely pathogenic<br>Likely oncogenic	Resistance/Reduced Sensitivity:<br>- Rebemadlin.<br>- MDM2 inhibitor AMGMDS2.
	TP53	Mutation – missense<br>p.Val173Leu exon 5/11	Evidence A<br>Pathogenic<br>Likely pathogenic<br>Likely oncogenic	Resistance/Reduced Sensitivity:<br>- Rebemadlin.<br>- MDM2 inhibitor AMGMDS2.
	TP53	Mutation – missense<br>p.Arg175His exon 5/11	Evidence A<br>Pathogenic<br>Oncogenic	Resistance/Reduced Sensitivity:<br>- Rebemadlin.<br>- MDM2 inhibitor AMGMDS2.
	TP53	Mutation – missense<br>p.Arg181Cys exon 5/11	Evidence A<br>Likely oncogenic	Resistance/Reduced Sensitivity:<br>- Rebemadlin.

			- MDM2 inhibitor AMGMDS2.
TP53	Mutation – missense p.Arg248Trp exon 7/11	Evidence A<br>Pathogenic<br>Likely oncogenic	Resistance/Reduced Sensitivity:<br>- Rebemadlin.<br>- MDM2 inhibitor AMGMDS2.
TP53	Mutation – missense p.Arg273Cys exon 8/11	Evidence A<br>Pathogenic<br>Likely pathogenic<br>Likely oncogenic	Resistance/Reduced Sensitivity:<br>- Rebemadlin.<br>- MDM2 inhibitor AMGMDS2.
TP53	Mutation – missense p.Arg282Gly exon 8/11	Evidence A<br>Pathogenic<br>Likely oncogenic	Resistance/Reduced Sensitivity:<br>- Rebemadlin.<br>- MDM2 inhibitor AMGMDS2.
TP53	Mutation – missense p.Glu286Lys exon 8/11	Evidence A<br>Pathogenic<br>Likely pathogenic<br>Likely oncogenic	Resistance/Reduced Sensitivity:<br>- Rebemadlin.<br>- MDM2 inhibitor AMGMDS2.
KRAS	Mutation – missense p.Thr20Ala exon 2/5	Evidence A<br>Poor outcome	Sensitivity/Response:<br>- Trametinib.<br>- Binimetinib.<br>- Cobimetinib.<br>- AZD5438.<br>- GDC-0623.
KRAS	Mutation – missense p.Leu19Phe exon 2/5	Evidence A<br>Oncogenic<br>Poor outcome	Sensitivity/Response:<br>- Trametinib.<br>- Binimetinib.<br>- Cobimetinib.<br>- AZD5438.<br>- GDC-0623.
NRAS	Mutation – missense p.Gln61Lys exon 3/7	Evidence A<br>Oncogenic	Sensitivity/Response:<br>- Trametinib.<br>- Metformin.
PIK3CA	Mutation – missense p.Ala1066Val exon 21/2	Evidence A<br>Likely oncogenic	Sensitivity/Response:<br>- Alpelisib.<br>- Capivasertib.<br>- RLY-2608.
PTEN	Mutation – frameshift p.Lys267ArgfsTer9 exon 7/9	Evidence A<br>Pathogenic	Sensitivity/Response:<br>- Ipatasertib.<br>- GSK26364771.<br>- AZD8186.
NF1	Mutation – frameshift p.Tyr930PhefsTer8 exon 21/5	Evidence B	Sensitivity/Response:<br>- Cobimetinib.<br>- Trametinib.

Note: It is important to underline an important number of alterations without a specific predictive value, but with clear functional relevance evidence of the genes APC, MSH6, MLH1, MUTYH, CDKN, CCND1, CCND3, FAS, FGF, RAC1, JAK2, HRAS, CTNNA1, RICTOR, RB1, and CIC.

Table S6. Actionable gene variants (distributed per ESCAT evidence tier) found for patients included in C4. For each variant, gene identification, nature of the alteration, functional relevance evidence for the alteration (A – Curated; B – Assumed; C - Predicted) and the

predictive value of the alteration is provided (2 - Investigational; 3 – Hypothetical target: Alteration-drug match is associated with antitumor activity, but magnitude of benefit is unknown (potential cancer-repurposing opportunity); 4 – Hypothetical target: pre-clinical evidence of actionability).

Tier	Gene	Alteration	Functional relevance evidence	Biomarker predictive value
2	MDM 2	Copy number alteration (Amplification)	Evidence A Oncogenic	Sensitivity/Response: - Brigimadlin. - Milademetan.
	TP53	Mutation – missense p.Pro151Thr exon 5/11	Evidence A Pathogenic Likely pathogenic Likely oncogenic	Sensitivity/Response: - Pazopanib. - Vorinostat.
	TP53	Mutation - stop gained p.Gln100Ter exon 4/11	Evidence A Pathogenic Likely pathogenic	Sensitivity/Response: - Pazopanib. - Vorinostat.
	TP53	Mutation – frameshift p.Arg213HisfsTer34 exon 6/11	Evidence B	Sensitivity/Response: - Pazopanib. - Vorinostat.
3	MDM2	Copy number alteration (Amplification)	Evidence A Oncogenic	Sensitivity/Response: - Brigimadlin. - Ezabenlimab.
	TP53	Mutation – missense p.Pro151Thr exon 5/11	Evidence A Pathogenic Likely pathogenic Likely oncogenic	Sensitivity/Response: - Azacytidine. - Eprenetapopt. - Chemotherapy. Resistance/Reduced Sensitivity: - RG7112.
	TP53	Mutation - stop gained p.Gln100Ter exon 4/11	Evidence A Pathogenic Likely pathogenic	Sensitivity/Response: - Azacytidine. - Eprenetapopt. - Chemotherapy. Resistance/Reduced Sensitivity: - RG7112.
	TP53	Mutation – frameshift p.Arg213HisfsTer34 exon 6/11	Evidence B	Sensitivity/Response: - Azacytidine. - Eprenetapopt. - Chemotherapy. Resistance/Reduced Sensitivity: - RG7112.
	MLH1	Mutation – missense p.Arg385His exon 12/19	Evidence C	Sensitivity/Response: - Talazoparib.
	BARD1	Mutation – missense p.Val713Met exon 11/11	Evidence C	Sensitivity/Response: - Olaparib.
4	CDK4	Copy number alteration (Amplification)	Evidence A Oncogenic	Sensitivity/Response: - Palbociclib. - Abemaciclib.
	MDM2	Copy number alteration (Amplification)	Evidence A Oncogenic	Sensitivity/Response: - Brigimadlin. - Milademetan.

	TP53	Mutation – missense p.Pro151Thr exon 5/1	Evidence A<br>Pathogenic<br>Likely pathogenic<br>Likely oncogenic	Resistance/Reduced Sensitivity:<br>- Rebemadlin.<br>- MDM2 inhibitor AMGMDS2.
	TP53	Mutation - stop gained p.Gln100Ter exon 4/11	Evidence A<br>Pathogenic<br>Likely pathogenic	Resistance/Reduced Sensitivity:<br>- Rebemadlin.<br>- MDM2 inhibitor AMGMDS2
	TP53	Mutation – frameshift p.Arg213HisfsTer34 exon 6/11	Evidence B	Resistance/Reduced Sensitivity:<br>- Rebemadlin.<br>- MDM2 inhibitor AMGMDS2.

Note: It is important to underline an important number of alterations without a specific predictive value, but with clear functional relevance evidence of the genes SDHD, IGF1R, CALR, NTRK1.

Table S7. Characteristics of the studies that also employed unsupervised consensus clustering to analyze data originated from STS molecular profiling approaches. This table provides, for each study, the STS histopathological subtypes of the samples that have been included, the types of molecular analyses that were performed (single or multi-omics, types of sequencing approaches that were used), the aims, methodological similarities and differences relative to our approach and, conceptually, the most relevant results.

Study Reference and Title	Samples	Molecular Analysis	Nature/ Aims	Methodological Approach	Results
[20]<br><br>"Integrative Clustering Reveals a Novel Subtype of Soft Tissue Sarcoma With Poor Prognosis"	247 STS samples<br><br>(including 56 DDLPS, 99 LMS,20 UPS)	Multi-omics:<br>RNA and miRNA sequencing.	<u>Nature</u> Exploratory.<br><br><u>Aims</u><br>1. To evaluate a potential new STS classification.<br><br>2. To clarify putative molecular mechanisms behind each of the identified clusters.	<u>Similarities</u><br>Use of consensus clustering.<br><br><u>Dissimilarities</u><br>1. Combination of consensus clustering with similarity network fusion.<br><br>2. Construction of a competing endogenous RNA network based on differentially expressed mRNAs, lncRNAs and miRNA.	1.Identification of 3 molecular clusters.<br><br>2. Correlation of one of the molecular clusters with worse prognosis.<br><br>3. Highlighting of a promising therapeutic target precisely for the cluster with the worse prognosis.
[41]<br><br>"Proteomic characterization identifies clinically relevant subgroups of soft tissue sarcoma"	272 Chinese patients with an STS<br><br>(including 35 DDLPS, 52 LMS and 43 UPS patients)	Single-omics:<br>Proteomics and phospho-proteomics<br><br>Both a proteomics (mass spectrometry-based) profiling and a phosphoproteomics (employing a Fe-NTA	<u>Aims</u><br>1. To unveil similarities and differences of the proteomic and phospho-proteomic profile of the included STS subtypes.	<u>Similarities</u><br>Use of consensus clustering.<br><br><u>Dissimilarities</u><br>1. Concomitant use of 2 different types of unsupervised clustering	1. Similitude of proteomic characteristics between angiosarcoma and epithelial sarcoma (by hierarchical clustering).

	phosphopeptides enrichment technology) analysis were performed using samples (both tumor samples and matched tumor-adjacent tissues samples – to permit the study of the immune microenvironment).	2. To uncover potential mechanisms of STS metastasis.<br><br>3. To identify STS immune microenvironment features.	(hierarchical and consensus) for different purposes.	2. Correlation of a high expression of SHC1 in angiosarcoma and epithelioid sarcoma with poor prognosis.<br><br>3. Identification of 3 proteomic clusters with various driven pathways and different clinical outcomes (by consensus clustering).<br><br>4. APEX1 and NPM1 promote, in the proteomic cluster portrayed by high cell proliferation rate, cell proliferation and drive the progression of cancer cells.<br><br>5. Highlighting of 3 immune subtypes with different tumor microenvironments (by consensus clustering).<br><br>6. Establishment of a potential association between immune evasion markers and metastasis development in STS.

Table S8. Most relevant distinctive molecular features of each transcriptomic cluster and conceptual rarity or novelty/originality of each feature.

**Transcriptomic Cluster**	**Distinctive Molecular Features**	**Rarity/ Originality**	**Additional Notes**
**1**	1. Over expression of CDK4 gene (possibly in correlation with an enrichment of this cluster in DDLPS, a subtype associated with CDK4 overexpression [34]).	<u>**Rarity**</u><br>1. Over expression of different genes involved in HRR in an STS cluster.<br>Alterations in genes involved in the HRR genes is a relatively	The coexistence of these molecular features in this cluster may either represent an additive effect of the molecular contribution from sarcoma samples labelled as DDLPS

	2. Under expression of different genes (BRCA1, BRCA2, FANCD2, PALB2, RAD51, CHEK1, and BRIP1) involved in HRR.	rare event in sarcomas. In a cohort of 7494 samples of soft tissue, bone and other sarcomas, only 2.5% of them harbored alterations in the homologous recombination repair pathways [34]. Another study reported that 4, 22, 21, 21 and 17% of sarcoma patients carry mutations in BRCA1, BRCA2, MDM2, PTEN and RAD1 respectively, and highlighted a subset of sarcomas that display high HRD [42].<br><br>**<u>Originality</u>**<br>1. Coexistence of CDK4 over expression and HRR genes under expression in an STS cluster.	(contributing with the CDK4 overexpression) and from sarcoma samples labelled as UPS and LMS (and also, potentially DDLPS) (contributing with the under expression of genes involved in HRR), or represent a new and previously undescribed sarcoma molecular subtype where these alterations concur.
**2**	<u>Over expression of cancer testis antigens (CTA).</u><br><br>1. Over expression of MAGE (MAGE-A12, MAGE-A2B, MAGE-A3, MAGE-B1, MAGE-B2, MAGE-C2) genes.<br><br>2. Over expression of SSX (SSX-1, SSX-2, SSX-2B and SSX-3) genes.	**<u>Rarity</u>**<br>1. Over expression of CTA in a cluster composed by samples of specific histopathological subtypes (DDLPS, LMS, UPS). Typically, the STS histological subtypes in which a higher expression of CTA (specifically MAGE, SSX, NY-ESO and PRAME) is found are synovial sarcomas and myxoid round cell liposarcomas [43,44,45].<br><br>2. Over expression of SSX genes in an STS cluster composed by DDLMS, LMS and UPS samples.<br>The related SSX genes, SSX1 and SSX2, are fusion partners of SYT, integrating the synovial sarcoma pathognomonic translocation and the resulting fusion protein SYT-SSX (the expression (mRNA) of SYT-SSX is detected in 89-100% of either monophasic or biphasic synovial sarcoma cases) [45,46]. But SSX-genes may also be highly expressed in other STS subtypes, namely in LMS (mainly SSX-1), uterine LMS (SSX-1, SSX-2 and SSX-4),	

	liposarcoma (mainly SSX-1, SSX-2 and SSX-3) and malignant fibrous histiocytoma (mainly SSX-2 and SSX-5) [46]. Interestingly, a significant fraction of STS samples co-express more than one SSX family member [46]. Another study reported a significant SSX mRNA expression in osteosarcoma, malignant peripheral nerve sheath tumor, malignant fibrous histiocytoma, liposarcoma, myxoid liposarcoma and LMS samples [47].<br><br>**Originality**<br>1. Over expression of MAGE-A12, MAGE-A2B, MAGE-A3, MAGE-B1, MAGE-B2, MAGE-C2 genes in an STS cluster.<br>Among the genes that encode the MAGE-I family of CTA (consisting of MAGE-A, MAGE-B and MAGE-C), MAGE-A4 is the most commonly overexpressed gene in STS, being highly expressed in synovial sarcoma (90% mRNA and 82-83% protein levels) and myxoid/round cell liposarcoma (68% at the protein level) [44,45]. Other studies report a high expression of MAGE-A1,-A2 and -A3 in osteosarcomas, but not in any STS [44].<br><br>2. Co-over expression of MAGE genes (MAGE-A12, MAGE-A2B, MAGE-A3, MAGE-B1, MAGE-B2, MAGE-C2) different from the up mentioned MAGE-A4, and SSX-1, SSX-2, SSX-2B and SSX-3 genes. Co-expression of MAGE and SSX has already been reported in colorectal cancer (and was found to be directly correlated with the development of metastasis to	

		the liver [48]), but has never been clearly reported in the STS subtypes that compose our cohort.	
**3**	1. Over expression of MHC class II/ HLA class II (HLA-DMA, HLA-DMB, HLA-DOA, HLA-DQA, HLA-DRA and HLA-DRB1) genes.<br><br>2. Under expression of CDKN (CDKN1C and CDKN2A) genes.<br><br>3. Under expression of FGFR (FGFR2 and FGFR3) genes.	**Originality**<br>1. Over expression of different genes that encode MHC/HLA class II peptides in STS.<br>In STS, the over expression of HLA class I genes (namely HLA-A) has been described in biphasic components of biphasic synovial sarcoma [49]. Another study looked into the genomic data of the neoplasms of 576 pediatric patients (262 of them with soft-tissue and bone sarcomas (rhabdomyosarcoma; non-rhabdomyosarcoma soft-tissue sarcoma; osteosarcoma; Ewing sarcoma)) with recurrent or refractory solid cancers enrolled in the MOSCATO-01 and the MAPPYACTS trials. This study unraveled a high HLA class I antigen expression in 27.1% of sarcoma samples, elevated frequencies of high HLA class I-positive samples in Ewing sarcoma, and osteosarcoma samples, high HLA-DR expression only in single specimens of osteosarcoma and an absence of HLA-DR expression in 73.4% of all sarcoma samples, more prominently in rhabdomyosarcoma samples [50]. Indeed, the over expression of genes that encode HLA class II peptides in STS has not been previously reported. The expression of HLA Class II peptides has been described in an array of human neoplasms and despite being constitutively expressed on professional antigen-presenting cells (pAPCs) (such as dendritic cells, macrophages and B cells), they may also be	The over expression of genes that encode different HLA class II peptides is a new finding in STS populations and may identify an STS cluster potentially more densely infiltrated by TIL, more prone to form TLS and characterized by enhanced antitumor immunity.

expressed either by other cell types in the tumor microenvironment (such as antigen-presenting cancer-associated fibroblasts and lymphatic endothelial cells) or by tumor cells (the so-called tumor-specific HLA-II (tsHLA-II) [51]. Different studies in a variety of cancer types highlighted a correlation between high expression of tsHLA-II and favorable prognosis (improved PFS and OS), and between high expression of tsHLA-II and increased levels of both CD4+ and CD8+ tumor-infiltrating lymphocytes (TIL), absence of lymphovascular invasion, increased formation of tertiary lymphoid structures (TLS), upregulation of genes associated with IFNg pathway activation, and higher levels of a plethora of different cytokines [51].

2. Under expression of FGFR genes in an STS cluster. Aberrations in components of the FGFR signaling pathway have been highlighted in an array of different sarcoma subtypes, most notably gastrointestinal stromal tumors, rhabdomyosarcomas, and liposarcomas [52]. These alterations comprise genetic events such as translocations, mutations, and amplifications as well as transcriptional overexpression, but lead, in the great majority of the cases, to overexpression and not under expression of FGFR [52].

3. Coexistence of over expression of HLA class II genes, and under expression of CDKN2A and FGFR genes in an STS cluster.

**4**	1. Over expression of CLDN 4 gene.<br><br>2. Over expression of genes that encode different structural proteins.	**Originality**<br>Claudins are a multigene family of proteins that are crucial elements of epithelial cell tight junctions, usually mediating cell–cell adhesion and selectively permitting the paracellular flux of ions and small molecules between cells [54]. They were recently brought to the spotlight as claudin 18.2, typically overexpressed in a subset of gastroesophageal neoplasms, became a compelling target for the either monoclonal antibodies (such as Zolbetuximab), whose use has provenly shown efficacy and survival advantage in phase III trials (such as SPOTLIGHT and GLOW), and also CAR-T cells and antibody-drug conjugates, whose use is currently being tested purely in an investigational context [54]. Claudin 4, more specifically, is typically over expressed in different epithelial malignancies types (namely breast, ovarian, lung, cervical, prostate, gastric and colorectal cancers), although its increased expression has not been previously described for any type of mesenchymal malignancy like STS (which is expected, considering that they are part of structures typically found in epithelia) [55].	In ovarian cancer, the over expression of the claudin 4 is associated with worse prognosis (shorter OS), even though it does not impact the degree of sensitivity to platinum [56].<br>In line with this findings, flourishing and prolific investigation on molecular therapies targeting claudin 4 has emerged, namely anti-claudin 4 extracellular domain antibodies, claudin 4 gene knockdown, clostridium perfringens enterotoxin (CPE), and C-terminus domain of CPE (C-CPE) [57].<br><br>This cluster is molecularly enriched in features that may reflect an increased transmembrane transportation and cell proliferative activity, which may characterize this molecular subset of STS.
